# Deep Insight of Design, Mechanism, and Cancer Theranostic Strategy of Nanozymes

**DOI:** 10.1007/s40820-023-01224-0

**Published:** 2023-11-21

**Authors:** Lu Yang, Shuming Dong, Shili Gai, Dan Yang, He Ding, Lili Feng, Guixin Yang, Ziaur Rehman, Piaoping Yang

**Affiliations:** 1 Key Laboratory of Superlight Materials and Surface Technology, Ministry of Education, College of Materials Science and Chemical Engineering, Harbin Engineering University, Harbin, 150001 People’s Republic of China; 2 Yantai Research Institute, Harbin Engineering University, Yantai, 264000 People’s Republic of China; 3 Key Laboratory of Green Chemical Engineering and Technology of Heilongjiang Province, College of Material Science and Chemical Engineering, Harbin University of Science and Technology, Harbin, 150040 People’s Republic of China; 4 Department of Chemistry, Quaid-i-Azam University, Islamabad, 45320 Pakistan

**Keywords:** Nanozymes, Classification, Prediction and design, Catalytic mechanism, Tumor theranostics

## Abstract

Since the discovery of enzyme-like activity of Fe_3_O_4_ nanoparticles in 2007, nanozymes are becoming the promising substitutes for natural enzymes due to their advantages of high catalytic activity, low cost, mild reaction conditions, good stability, and suitable for large-scale production. Recently, with the cross fusion of nanomedicine and nanocatalysis, nanozyme-based theranostic strategies attract great attention, since the enzymatic reactions can be triggered in the tumor microenvironment to achieve good curative effect with substrate specificity and low side effects. Thus, various nanozymes have been developed and used for tumor therapy. In this review, more than 270 research articles are discussed systematically to present progress in the past five years. First, the discovery and development of nanozymes are summarized. Second, classification and catalytic mechanism of nanozymes are discussed. Third, activity prediction and rational design of nanozymes are focused by highlighting the methods of density functional theory, machine learning, biomimetic and chemical design. Then, synergistic theranostic strategy of nanozymes are introduced. Finally, current challenges and future prospects of nanozymes used for tumor theranostic are outlined, including selectivity, biosafety, repeatability and stability, in-depth catalytic mechanism, predicting and evaluating activities.

## Highlights

- Classification and catalytic mechanism of nanozymes with different mimicking activities are dissertated.
- Activity prediction and rational design methods of nanozymes are highlighted, including density functional theory, machine learning, biomimetic and chemical design.
- The roles of nanozymes in different synergistic theranostic strategies for tumor are summarized and explained by representative examples in the past five years.

## Graphical abstract

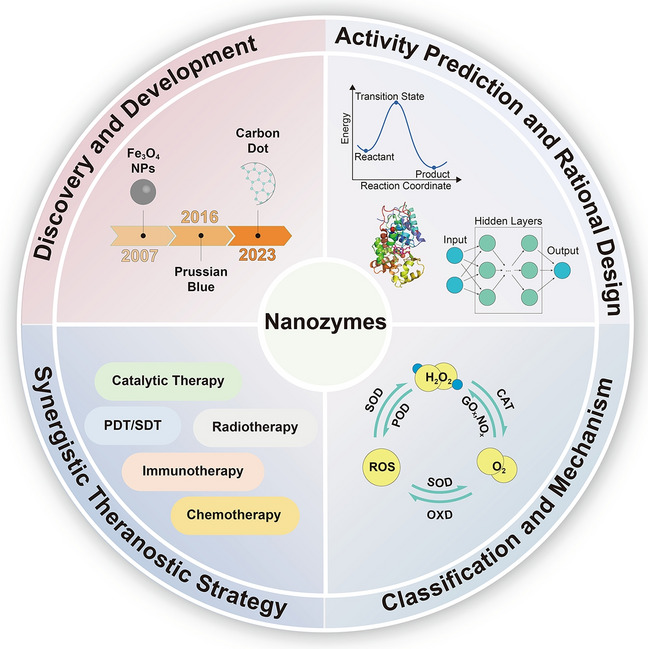

## Introduction

According to data from the International Agency for Research on Cancer, in 2020, there were 19.29 million new cancer cases worldwide, with 9.96 million deaths. Breast cancer was becoming the most common type of cancer, with number about 2.3 million new cases (11.7% of the total), followed by lung cancer (11.4%), colorectal cancer (10.0%), prostate cancer (7.3%) and gastric cancer (5.6%). Nevertheless, lung cancer remains the main culprit in cancer deaths, with approximately 1.8 million people (18%) dying from it [1]. At present, the treatment strategies for cancer mainly include radiotherapy (RT), chemotherapy, and surgical resection, which often bring strong side effects to patients [2–5]. As emerging treatment methods, photodynamic therapy (PDT) and photothermal therapy (PTT) have become potential anti-tumor strategies due to their negligible invasiveness, high selectivity, and low toxicity [6–10]. However, due to the disadvantages of light penetration depth, tumor hypoxia, and the heat resistance of tumor cells, the further clinical application is limited [10–15]. Therefore, development of effective, easy to operate, and targeted treatment strategies has always been one of the key issues in the field of cancer prevention and treatment research. Developing and improving tumor treatment strategies and drugs to further prolong patient survival is an urgent goal for researchers both domestically and internationally.

In recent years, researchers have been committed to exploring tumor treatment strategies specifically activated by tumor microenvironment (TME), following the principle of “adaptation to local conditions, and adaptation to the situation”. Catalysis and medicine were considered as two unrelated research fields, and different scientific phenomena were studied separately. However, with the latest progress in the field of nanochemistry, a large number of new nanocatalysts have emerged, such as nanozyme [16], photocatalyst [17], and electrocatalyst [18]. These catalysts have been used in the body to start the catalytic reaction and regulate the biological microenvironment to achieve therapeutic effects. With the rapid development of nanocatalysts, a new concept called “nanocatalytic medicine” arose at the historic moment, defined as “the use of biocompatible nanomaterials for catalytic reaction-based disease diagnosis and treatment” [19].

Nanozymes are a kind of nanomaterial with biocatalytic function, which can catalyze the substrate of natural enzymes based on specific nanostructures and act as substitutes for enzymes. The discovery of nanozymes reveals for the first time that nanomaterials contain a unique biological effect of enzyme-like catalytic activity. Catalysis is a ubiquitous phenomenon in various physiological processes of organisms. It not only serves as the necessary response for maintaining basic human physiological activities, but also provides key mechanism guidance for the design of treatment strategies. A common method for the manufacturing of nanocatalytic drugs is to use wet chemical methods to in situ grow nanozymes (such as CeO_2_) in solution, and then perform appropriate surface engineering to endow them with biocompatibility and versatility. Another method is to synthesize various nanocarriers in a controllable manner, such as liposome, mesoporous silica, metal–organic framework (MOF), etc. [20–23], and then load molecular catalysts (such as enzymes) to construct composite nanocarriers. In addition, with recent achievements in the field of single-atom catalysis, higher catalytic efficiency can be achieved by immobilizing a series of transition metal atoms onto specific nanocarriers, further enriching the list of nanocatalytic drugs [24].

More importantly, nanozymes can fully utilize the TME to achieve targeted and specific therapy, because the TME refers to the tumor cells and their surrounding microenvironment. TME not only contains surrounding microvessels, fibroblasts, immune cells, lymphocytes, bone marrow-derived inflammatory cell signaling molecules, and other extracellular matrix, but also represents various biological characteristics within tumor tissue, such as low oxygen content, acidic pH value [25, 26], overexpressed H_2_O_2_ about 50 − 100 × 10^−6^ M [27–32], and redox environment with high glutathione (GSH) concentration of 10 × 10^−3^ M [33, 34]. Tumor hypoxia influences the therapeutic effect, and promotes tumor metastasis and proliferation. In addition, high concentrations of GSH in cells can deplete the generated reactive oxygen species (ROS), which will seriously affect the effectiveness of nanozyme-based therapy. Taking advantage of abnormal biochemical indicators in tumor tissue microenvironment and tumor cells is a potential direction for exploring and developing new nanozyme-based therapeutic models [35–38].

Accordingly, by introducing non-toxic or low-toxic nanozymes into pathological areas, therapeutic chemical reactions can be “catalyzed” in a more biocompatible and sustained manner. In these catalytic reactions, reactants are usually inherent biochemical substances in the pathological region, rather than delivered therapeutic agents. Thus, “nanocatalytic medicine” can be used to guide catalytic reactions and optimize treatment outcomes. In the past century, chemists strived to achieve efficient and selective catalysis, which can be cleverly transformed into high efficiency and low side effects in therapeutic diagnostics. In the past few years, this emerging catalytic therapy strategy has greatly promoted the progress of many nanomedicine fields, providing effective treatment effects for various pathological abnormalities, including cancer, bacterial infections, inflammation to brain injury and other diseases [39–42].

Recently, several excellent previous reviews have focused on the nanozyme from different perspectives. Fan et al. summarized the biorthogonal catalytic activity of transition metal catalysts [43], design of nanozymes by machine learning [44], and multifaceted nanozymes for synergistic antitumor therapy [45] by using three different review articles. Qu et al. emphasized the nanozyme progress before 2018, focusing on the classification, catalytic mechanisms, activity regulation, and applications, while the latest developments such as machine learning and theory calculations were not included [16]. Zhao et al. focused on the bio-applications of nanozymes, including cancer diagnosis and therapy [46]. Xu et al. described activity-regulating strategies of nanozymes for biomedical applications (i.e., doping, vacancy, modification, size, and morphology) [47]. Xu et al. introduced activities and biological applications of nanozymes [48]. To the best of our knowledge, there is no full-scale review of the development of nanozymes in the last five years, including deep insight of design (i.e., density functional theory (DFT), machine learning, biomimetic and chemical design), mechanism, and cancer theranostic strategy to further stimulate the motivation of developing novel nanozymes.

Based on the above discussion, herein, the latest research progress of nanozymes is introduced systematically. First, the discovery and development of nanozymes are introduced. Second, the classification and catalytic mechanism of peroxidase (POD), catalase (CAT), superoxide dismutase (SOD) and oxidase (OXD) mimicking nanozymes and hydrolytic nanozymes are summarized. Third, from the perspectives of DFT, machine learning, biomimetic design, and chemical design, the reasonable design criteria for predicting the activity of nanozymes are introduced. Fourth, the recent progress in the in vivo diagnosis and nanozyme-assisted synergistic treatment application of tumor are reviewed. Finally, the development prospects and main problems to be solved of nanozymes are summarized and prospected.

## Discovery and Development

When it comes to enzymes, people may first think of biologically active substances such as proteins and nucleic acids with catalytic effects. In 1926, biochemist Dr. James B. Sumner discovered the first enzyme (urease) and confirmed that it was a protein molecule. Since then, protein has been identified as the substance composition of all enzymes until the discovery of nucleases in 1982. On these grounds, natural enzymes are proteins or RNAs synthesized by living cells, which are important class of biocatalysts with high specificity and catalytic activity towards their substrates (reactants) [49–51]. Under mild conditions, they can efficiently promote chemical reactions within organisms, with a catalytic efficiency of approximately 107 to 1013 times than that of inorganic catalysts. Therefore, in actual production, they are often used to improve production efficiency [52–54]. However, enzymes are not perfect either. The chemical essence of enzymes is proteins or RNA, which have complex spatial structures. At the same time, the spatial structure of enzymes is prone to damage under high temperatures, strong acids, strong alkalis, and other environments, resulting in loss of activity. The strict requirements for storage environment make it difficult for enzymes to be stored for a long time [55, 56].

**Fig. 1:**
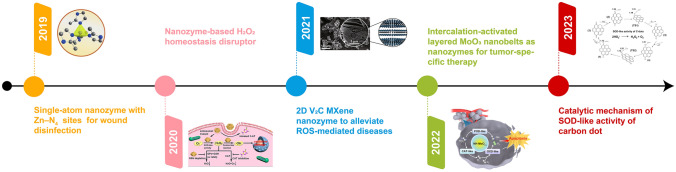
A brief timeline for the evolution of nanozymes in recent five years of 2019 [61], 2020 [62], 2021 [63], 2022 [64], and 2023 [65]. Reproduced with permissions. Copyright 2019, Wiley–VCH Verlag GmbH & Co. KGaA, Weinheim. Copyright 2020, American Chemical Society. Copyright 2021, Springer Nature. Copyright 2022, Wiley–VCH GmbH. Copyright 2023, Springer Nature

Nanozymes are nanomaterials with inherent enzyme-like activity that can effectively catalyze substrate conversion under physiological conditions, also following the same kinetic mechanisms as natural enzymes [55–58]. In 2004, nanozyme was first coined to describe the transphosphorylation reactivity of triazacyclononane-modified gold nanoparticles by Pasquato and co-workers [59]. In an accidental experiment in 2007, the group of Yan found that when the size of Fe_4_O_3_ was small to nanometer, an unexpected catalytic activity similar to “natural enzyme” appeared, namely, a catalytic activity similar to horseradish peroxidase (HRP) presented [60]. In this article, the POD-like activity of Fe_3_O_4_ nanoparticles toward typical POD substrates was reported. Researchers confirmed the universal pattern of catalytic activity from nanomaterials, and named them “nanozymes”. This discovery breaks the long-standing understanding among researchers that inorganic materials are biologically inert materials. This study reveals the inherent biological effects and new characteristics of nanomaterials, expanding the concept of “nano-effects” from the fields of light, sound, and electricity to the field of “enzyme-like catalysis” biological effects. Compared with natural enzymes, nanozymes have several advantages of low cost, good stability, high catalytic activity, mild reaction conditions, and easy for large-scale production. Since then, nanozymes have entered the researchers’ vision. Wei et al*.* summarized a brief timeline for the evolution of natural enzymes and nanozymes with key points from 1946 to 2018 [58], following that, Fig. 1 shows the evolution of nanozymes in recent five years [61–65], indicating the fast development of nanozymes.

In order to meet the demand of basic research, application research, and industrial promotion of nanozymes, scientists have established a standard that can quantitatively compare the catalytic activity and kinetics of various nanozymes, and defined the catalytic activity unit (Nanozyme unit, U) of nanozymes. Namely, under optimal reaction conditions, the amount of nanozyme required for the catalysis of 1 μmol substrate into product per minute is 1 U. The specific activity (U mg^−1^) of nanozyme is defined as the catalytic activity of a unit mass of nanozyme. The specific activity detection conditions and calculation formulas of nanozyme are further specified, which further promote the development of detection and diagnostic technologies based on nanozyme [60]. In recent years, Chinese scientists pioneered a new strategy of “nanozyme-catalyzed tumor therapy” through the regulation of in vivo activity of nanozymes [19]. In addition, a nanozyme-catalyzed photoacoustic (PA) imaging technology was successfully developed by constructing a type of nanozymes [66]. The results show that the versatility and effects of nanozymes open up many new potential applications for biotechnology, medicine and environmental chemistry. The International Union of Pure and Applied Chemistry listed nanozymes as one of the “Top Ten Emerging Technologies in Chemistry in 2022”.

## Classification and Catalytic Mechanism of Nanozymes

So far, a large number of nanomaterials including metal oxide nanoparticles [67, 68], carbon-based nanomaterials [69, 70], noble metal nanoparticles [71], and MOFs [72, 73], have been proven to be able to play a catalytic role like natural enzymes. Based on these new characteristics, nanozymes were widely used for virus detection, biosensing, environmental governance, cancer diagnosis and treatment, antibacterial, and cellular protection of intracellular biomolecules [74–76]. Normally, based on catalytic mechanisms, enzymes can be divided into seven types of oxidoreductase, hydrolase, transferase, isomerase, lyase, ligase, and transposase. Most nanozymes can simulate the activity of oxidoreductases, such as SOD, POD, CAT, and OXD, besides, only a small portion has catalytic abilities similar to hydrolases or other enzymes [77]. Representative Michaelis–Menten constant (*K*_m_) and maximum reaction rate (*V*_max_) of previously reported nanozymes were summarized in Table 1.

**Table 1:** Representative *K*_m_ and *V*_max_ values of previously reported nanozymes

Nanozymes	Catalytic activities	Substrates	Outcomes	Applications	Refs.
HRP (Natural enzymes for comparison)	POD-like	TMB (3,3',5,5'-tetramethylbenzidine)	*V*_m_ = 10.00 × 10^–8^ M s^–1^ *K*m = 0.434 mM	Immunoassays	[60]
		H_2_O_2_	*V*_m_ = 8.71 × 10^–8^ M s^–1^ *K*m = 3.70 mM		
Fe_3_O_4_ MNPs	POD-like	TMB	*V*_m_ = 3.44 × 10^–8^ M s^–1^ *K*m = 0.098 mM		
		H_2_O_2_	*V*_m_ = 9.78 × 10^–8^ M s^–1^ *K*m = 154 mM		
HRP	CAT-like	H_2_O_2_	*V*_m_ = 0.689 nM s^–1^ *K*m = 10.35 mM	–	[78]
His-Fe_3_O_4_	CAT-like	H_2_O_2_	*V*_m_ = 5.28 nM s^–1^ *K*m = 37.99 mM		
ZnCr_2_O_4_	POD-like	H_2_O_2_	*V*_m_ = 1.3751 × 10^–8^ M s^–1^ *K*m = 8.20 mM	–	[79]
ZnMn_2_O_4_	POD-like	H_2_O_2_	*V*_m_ = 2.5295 × 10^–8^ M s^–1^ *K*m = 204.79 mM		
ZnFe_2_O_4_	POD-like	H_2_O_2_	*V*_m_ = 8.6960 × 10^–8^ M s^–1^ *K*m = 170.94 mM		
LiCo_2_O_4_	POD-like	H_2_O_2_	*V*_m_ = 128.63 × 10^–8^ M s^–1^ *K*m = 265.84 mM		
CuO	POD-like	H_2_O_2_	*V*_m_ = 16.1 × 10^–8^ M s^–1^ *K*m = 0.40 M	Glucose and antioxidant detection	[80]
		TMB	*V*_m_ = 10.49 × 10^–8^ M s^–1^ *K*m = 0.025 M		
FeWO_X_ NSs	POD-like	TMB	*V*_m_ = 12.25 × 10^–8^ M s^–1^ *K*m = 0.03 × 10^–3^ M	Cancer sensing	[81]
		H_2_O_2_	*V*_m_ = 435 × 10^–8^ M s^–1^ *K*m = 3.26 × 10^–3^ M		
PtFe@ Fe_3_O_4_	POD-like	TMB	*V*_m_ = 5.477 × 10^–8^ M s^–1^ *K*m = 0.213 mM	Tumor catalytic therapy	[82]
		H_2_O_2_	*V*_m_ = 1.078 × 10^–7^ M s^–1^ *K*m = 53.55 mM		
PtFe	POD-like	TMB	*V*_m_ = 6.02 × 10^–8^ M s^–1^ *K*m = 0.237 mM		
		H_2_O_2_	*V*_m_ = 8.182 × 10^–8^ M s^–1^ *K*m = 217.6 mM		
PVP-IrNPs	CAT-like	H_2_O_2_	*V*_m_ = 540 × 10^–6^ M s^–1^ *K*m = 297 mM	Cellular protective effect	[83]
	POD-like	H_2_O_2_	*V*_m_ = 0.385 × 10^–6^ M s^–1^ m = 266 mM		
Cit-IrNPs	POD-like	TMB	*V*_m_ = 1.7 × 10^–6^ M s^–1^ *K*m = 0.0906 mM	Selective oxidation of aromatic alcohols	[84]
Pt NPs	POD	TMB	*V*_m_ = 3.02 × 10^–8^ M s^–1^ *K*m = 1.78 × 10^–3^ mM	Rapid test	[85]
Au@Rh-ICG-CM	CAT-like	H_2_O_2_	*V*_m_ = 72.5 mM min^–1^ *K*m = 509.9 mM	Bimodal imaging and enhanced PDT	[86]
Pt@HP@M	CAT	H_2_O_2_	*V*_m_ = 3.56 × 10^–7^ M min^–1^ *K*m = 40.1 × 10^–3^ M	Sonodynamic therapy	[87]
	POD	TMB	*V*_m_ = 5.66 × 10^–6^ M min^–1^ *K*m = 1.628 × 10^–5^ M		
P-RuCu	POD-like	H_2_O_2_	*V*_m_ = 81.7 nM s^–1^ *K*m = 0.25 nM	Catalytic therapy and RT	[88]
PdPtAu MNPs	POD	H_2_O_2_	*V*_m_ = 4.96 × 10^−7^ M s^–1^ *K*m = 2.35 × 10^−3^ M	Radioimmunotherapy	[89]
FeN-carbon hollow structure	OXD-like	H_2_O_2_	*V*_m_ = 45.27 nM s^–1^ *K*m = 0.55 mM	Nanozymes therapy	[90]
	POD-like	H_2_O_2_	*V*_m_ = 567.9 nM s^–1^ *K*m = 175.5 mM		
		TMB	*V*_m_ = 299.8 nM s^–1^ *K*m = 0.18 mM		
	UOD-like	Uric acid	*V*_m_ = 1.36 nM min^–1^ *K*m = 16.64 μM		
	CAT-like	H_2_O_2_	*V*_m_ = 2.34 mg L^–1^ min^–1^ *K*m = 0.09 M		
N-PCNSs-3	SOD-like	TMB	*V*_m_ = 0.42 × 10^–8^ M s^–1^ *K*m = 0.084 mM	Tumor catalytic therapy	[91]
N-PCNSs-5	SOD-like	TMB	*V*_m_ = 0.27 × 10^–8^ M s^–1^ *K*m = 0.095 mM		
FeN_3_P-SAzyme	POD-like	TMB	*V*_m_ = 4.65 × 10^–5^ M min^–1^ *K*m = 2.06 × 10^–3^ mM	Nanozymes catalytic therapy	[92]
FeN_4_-SAzyme	POD-like	TMB	*V*_m_ = 2.16 × 10^–5^ M min^–1^ *K*m = 1.07 × 10^–3^ mM		
Fe_3_O_4_ nanozyme	POD-like	TMB	*V*_m_ = 3.34 × 10^–5^ M min^–1^ *K*m = 3.49 × 10^–2^ mM		
HRP	POD-like	TMB	*V*_m_ = 6.37 × 10^–5^ M s^–1^ *K*m = 5.55 mM		
Cu_2-x_Te	POD-like	TMB	*V*_m_ = 7.3 × 10^–7^ M s^–1^ *K*m = 189 mM	Antitumor immunotherapy	[93]
	GSHOx-like	GSH	*V*_m_ = 1.49 × 10^–5^ M s^–1^ *K*m = 0.29 mM		
CdCo_2_O_4_ NSs	POD-like	H_2_O_2_	*V*_m_ = 3.75 × 10^–8^ M s^–1^ *K*m = 0.325 mM	Colorimetric assay of glucose	[94]
GPX-mimicking MIL-47(V)-X	SOD-like	H_2_O_2_	*V*_m_ = 0.0019 M s^–1^ *K*m = 0.003 mM	Nanozymes catalytic therapy	[95]
		GSH	*V*_m_ = 0.0035 M s^–1^ *K*m = 2.85 mM		
CAT (Natural enzymes for comparison)	CAT	Glycolic acid	*V*_m_ = 3.0 μM s^–1^ *K*m = 249.4 mM	Solving the H_2_O_2_ by-product problem	[96]
Co-ordination polymers	CAT-like	Glycolic acid	*V*_m_ = 2.4 μM s^–1^ *K*m = 112.2 mM		

### POD-like Nanozymes

POD is a family of enzymes which has the ability to catalyze biological reactions. Peroxides, mainly including hydrogen peroxide and lipid peroxide, are reduced during the catalytic reaction, meanwhile, the redox substrate can be oxidized as an electron donor. POD typically catalyzes substrate oxidation by consuming H_2_O_2_ or organic peroxides [97, 98]. Most natural PODs are hemeproteins that can activate H_2_O_2_ to generate intermediate substances with high valence states, enabling the extraction of electrons from different substrates. Therefore, various iron-based nanomaterials have been reported to possess POD-like activities. For instance, the pioneering work of Yan and co-workers in 2007 showed that Fe_3_O_4_ nanoparticles have inherent catalytic activity for classic POD substrates, thus opening the door to develop POD-like nanomaterials [60]. All the nanomaterials of magnetic nanoparticles (Fe_3_O_4_, CoFe_2_O_4_, ZnFe_2_O_4_, etc.), metal oxide nanomaterials (Co_3_O_4_, CeO_2_, MnO_2_, etc.), carbon nanomaterials (carbon nanodot, carbon nanotube (CNT), graphite oxide (GO), reduced graphite oxide (RGO), fullerene-C_60_, carbon nitride dot, etc.), metal sulfide nanomaterials (ZnS, MoS_2_, etc.), metal nanomaterials (Au, Ag, Pt, etc.) and nanocomposites (GO-Fe_3_O_4_, Cu-Ag/RGO, MoS_2_/GO, etc.) have been found to have inherent POD-like activity.

**Fig. 2:**
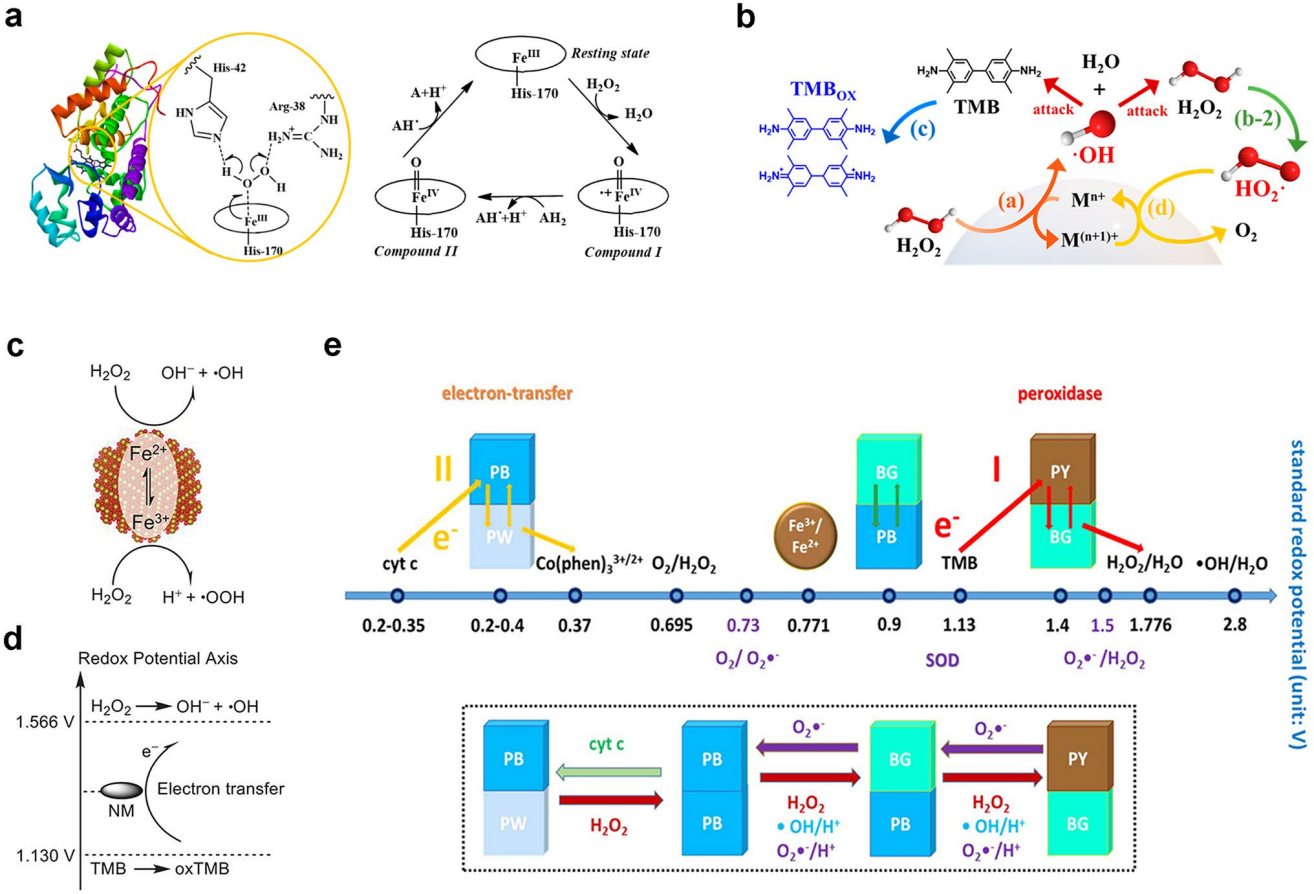
**a** Schematic diagram of HRP reaction pocket and corresponding catalytic cycle. **b** Schematic diagram of metal oxide as a POD-like material. Reproduced with permission [99]. Copyright 2021, American Chemical Society.** c**, **d** Mechanisms of POD-like nanozymes [101]. Reproduced with permission. Copyright 2023, Wiley–VCH GmbH. **e** Schematic diagram of the multienzyme-like activity and mechanism of PBNPs based on standard redox potentials [102]. Reproduced with permission. Copyright 2016, American Chemical Society

Recently, people devoted to gaining a deep understanding of the catalytic mechanism of POD-like nanozymes. Prior to this, analysis the composition, structure, and catalytic mechanism of natural HRP is necessary to reveal the POD-like mechanism of nanozymes. As reported, Fe(III)-protoporphyrin IX composed of His-170 as proximal ligand is the active site of HRP (Fig. 2a) [99, 100]. In the reaction pocket, His-42 and Arg-38 amino acids also play a crucial role in the O − O bond activation of targeted and adsorbed H_2_O_2_. During the reaction process, H_2_O_2_ (reduced to H_2_O) first oxidizes Fe(III) (i.e. stationary state) to Fe(IV)-oxo, and retains ·OH radicals on the porphyrin ring (compound I). Then, when the two-electron donor (AH_2_) is present, the reactive intermediate can be reduced to Fe(IV)-oxo (compound II) and static Fe(III) by two-step reduction. In order to clarify the mechanism difference between HRP and metal oxide nanozyme, the metal oxide surface reaction was also revealed by Peng et al. as follows: the generation of ·OH and hydrogen peroxide radical (HO_2_·), oxidation of the chromogenic substrate, and surface regeneration of active site (Fig. 2b) [99]. The rate-limiting step in the reaction is the reduction of H_2_O_2_ to ·OH through the surface metal active sites. Subsequently, generated ·OH radical oxidates chromogenic substrates of 3,3',5,5'-tetramethylbenzidine (TMB). In addition, ·OH radical also oxidizes H_2_O_2_ to HO_2_· radical, then, the surface active site was re-generated by reducing M^(n+1)+^ to M^n+^ from HO_2_· radical. Therefore, Fe_3_O_4_ nanoparticles produce free ·OH radicals, while HRP retains the free radicals on the iron porphyrin ring. Peng et al. also pointed out that under the same concentration of nanozyme (based on the surface active site), larger Fe_3_O_4_ nanoparticles (~ 86 nm) have better catalytic activity than smaller Fe_3_O_4_ nanoparticles (~ 20 nm), which can be attributed to the higher Fe^2+^/Fe^3+^ ratio of the polycrystalline Fe_3_O_4_ nanoparticles.

In order to rationalize the sustainability of POD activity in Fe_3_O_4_ nanozymes, Zhang et al. revealed the internal atomic changes of Fe_3_O_4_ nanozymes by using X-ray photoelectron spectroscopy and X-ray absorption near edge structure [103]. After the oxidation of surface Fe^2+^ to Fe^3+^ and the generation of ·OH radicals, electrons migrate from the inner Fe^2+^ to the surface through the Fe^2+^-O-Fe^3+^ chain, thereby, achieving the regeneration of ·OH radicals. The POD-like mechanism of Fe_3_O_4_ nanozyme was summarized as follows. (1) Fenton-like reaction on the surface of particles happens. Namely, H_2_O_2_ receives electrons from Fe^2+^ on the surface and dissociates into highly active ·OH to oxidize the substrate. At the same time, the surface Fe^2+^ is oxidized to Fe^3+^. (2) Internal electron transfer occurs. The adjacent Fe^2+^ ions inside the particles transfer their own electrons to the surface Fe^3+^ through the Fe^2+^ − O − Fe^3+^ chain, resulting the regeneration of surface Fe^2+^, and providing power for the continuous reaction. (3) Excess Fe ions migrate outward. In order to maintain electrical neutrality, with the in situ oxidation of internal Fe^2+^, excess Fe^3+^ in the lattice migrates towards the surface, leaving cation vacancies. (4) Chemical composition is changed. As the reaction continues, Fe_3_O_4_ nanozymes undergo oxidation from the outside to the inside, ultimately, leading to a phase transition to γ-Fe_2_O_3_. The oxidation process of Fe_3_O_4_ nanozyme triggered by POD-like reaction is similar to the low-temperature air oxidation of traditional magnetite, in which Fe^3+^ migration is a rate-limiting step. Gao et al. revealed the interconversion process between Fe^2+^ and Fe^3+^ ions by Fenton reaction on Fe_3_O_4_ surfaces, which plays an important role in the catalysis reaction (Fig. 2c) [101]. In contrast, electron transfer mechanism was proposed for the POD-like activity of Co_3_O_4_ (Fig. 2d). Zhang and co-workers theorized the multienzyme-like activities of prussian blue nanoparticles (PBNPs), which can be explained as abundant redox potentials from different forms, and efficient electron transporters were generated (Fig. 2e) [102].

**Fig. 3:**
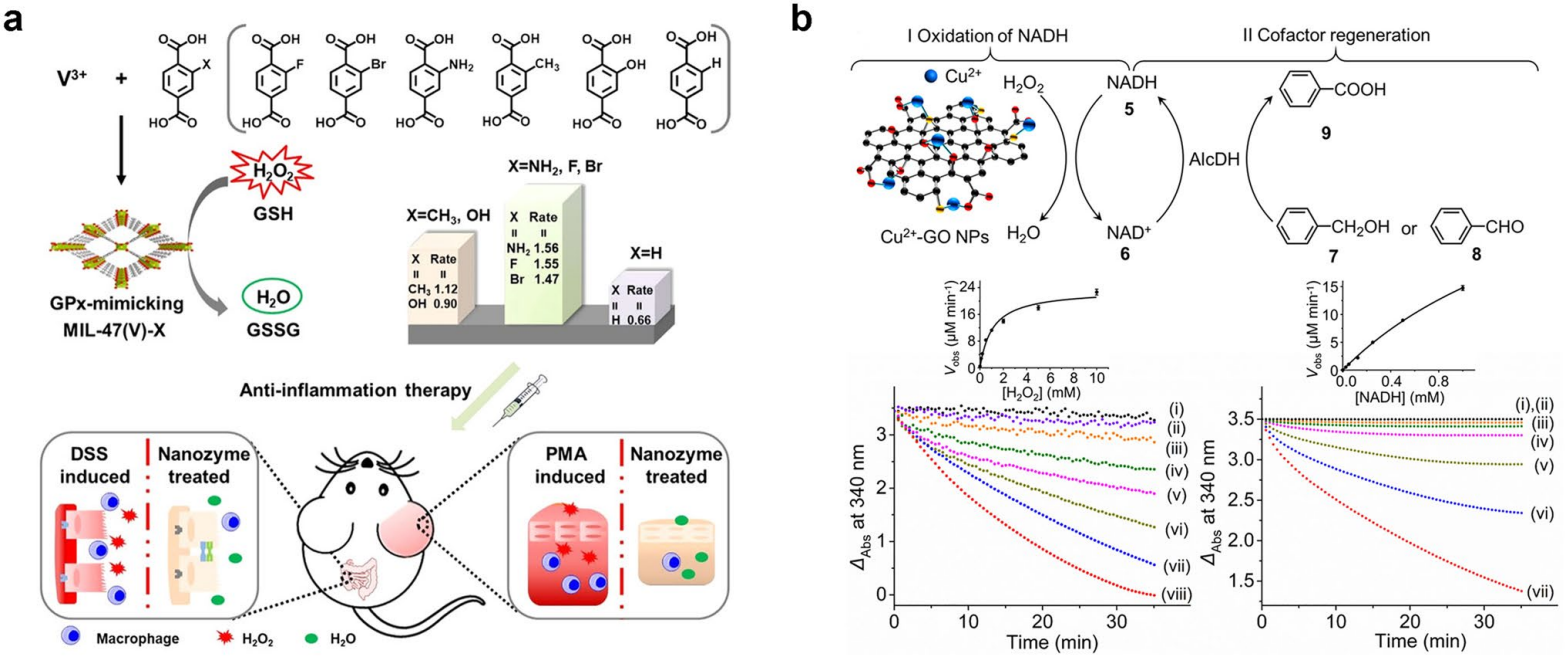
**a** Schematic synthesis diagram of the GPX-like MIL-47(V)-X nanozyme for anti-inflammatory treatment [95]. Reproduced with permission. Copyright 2020, Wiley-VCH Gmb. **b** Cu^2+^-GO nanoparticles with NADH POD-like activity for regeneration of NAD^+^ cofactor [105]. Reproduced with permission. Copyright 2017, American Chemical Society

In addition, as a member of the POD family, the natural glutathione peroxidase (GPX) in organisms can catalyze GSH to convert excess H_2_O_2_ into H_2_O, reducing its damage to the organism. Therefore, GPX has received widespread attention in numerous disease treatment studies [63, 104]. In recent years, the exploration of MOFs in simulating hydrolytic enzymes, POD, and other enzymes has provided new ideas for researchers [73]. In view of this, Wei et al. designed a ligand engineering strategy to regulate the GPX-like activity of MOF nanozymes [95]. The electronic properties of 1,4-benzoic acid ligands were adjusted by replacing H with OH, NH_2_, CH_3_, F, and Br, respectively. Among these isomorphic MIL-47(V)-X, the MIL-47(V)-NH_2_ exhibits the highest GPX-like activity. It has been demonstrated that its excellent antioxidant ability in vitro can decrease ROS levels and prevent ear inflammation and colitis in the body (Fig. 3a). Willner et al. prepared GO nanoparticles about 6 nm by using the modified Hummers method, and then solvothermal treatment with dimethylformamide was combined to synthesize nitrogen-doped graphene nanoparticles. After further reaction with CuCl_2_, Cu^2+^-GO nanoparticles were obtained [105]. This nanozyme can simulate the activity of reduced nicotinamide adenine dinucleotide (NADH) peroxidase (NADH-like POD). Cu^2+^-GO nanoparticles have the catalysis ability to oxidate NADH by using H_2_O_2_ as a biologically active NAD^+^ cofactor. The catalytic activity was further explored through a steady-state kinetic study, which provides a new NAD^+^ cofactor regeneration process that can be used to drive biocatalytic conversion (Fig. 3b).

Because of the mixed valence states of Ce^3+^, Ce^4+^, and the formation of oxygen vacancies, CeO_2_ exhibits excellent catalytic performance in various applications [106–109]. Compared with natural enzymes, CeO_2_ nanozymes can exhibit Ce^3+^-dependent POD-like activity over a wide pH and temperature range through Fenton-like reactions [107, 110]. Notably, the POD-like activity of CeO_2_ nanozymes is highly dependent on the ratio of Ce^3+^/Ce^4+^ [111]. By adjusting the Ce^3+^/Ce^4+^ ratio and designing more oxygen vacancies, the POD-like activity of CeO_2_ can be improved. Usually, the proportion of Ce^3+^ ions increases with a decrease in particle size [112]. Therefore, the controllable synthesis of ultra-small CeO_2_ nanozymes may be one of the ways to enhance their POD-like activity. We report a cerium-based nanozyme with thermally enhanced dual-enzyme activity and GSH depletion ability for efficient catalytic tumor therapy [113]. Firstly, Bi_2_S_3_ nanorod was coated by dendritic mesoporous silica (DMSN), the ultra-small CeO_2_ was loaded into the dendritic mesopores to prevent the aggregation of CeO_2_, and polyethylene glycol (PEG) modification was used to prepare the cerium-based PEG/Ce-Bi@DMSN nanozyme. We chose an improved reverse micelle method using cerium acetate hydrate as precursor to prepare ultra-small CeO_2_ nanozymes about 3 − 4 nm, which showed a high Ce^3+^/Ce^4+^ ratio of 1.94:1. The nanozymes exhibit CAT-like activity, POD-like activity, and GSH depletion ability in weakly acidic environments, achieving high toxicity ·OH accumulation while alleviating tumor hypoxia and weakening the reducibility of TME.

### OXD-like Nanozymes

As well known, OXD aroused widespread interest due to its crucial role in the evolution of aerobic organisms through the selective and effective utilization of O_2_ oxidant. In the biosphere, the superfamily of heme-copper oxidase, such as cytochrome c oxidase, can catalyze and consume most O_2_ [114]. Meanwhile, OXD exhibits extensive applications in industries such as pharmaceuticals, food, and biotechnology. As electron acceptor, when the O_2_ is present, the oxidation of the substrate (electron donor) can be catalyzed to generate corresponding oxidation products, H_2_O and H_2_O_2_. Recently, researchers found that lots of inorganic nanomaterials possess the ability to catalyze the oxidation of one or more substrates with assistance by O_2_, exhibiting OXD-like activity, such as cerium-based oxides, noble metals (Au, Pt, Pd, etc.), manganese-based oxides, MOFs, and so on [115–118]. According to the oxidized groups of the substrates, OXD-like nanozymes have been classified as glucose oxidase (GOx)-like, polyphenol oxidase-like, cytochrome c oxidase-like, sulfite oxidase-like, etc. [119].

Nanozymes with OXD-like activity can catalyze the oxidation process on substrates, and the catalytic process accompanied by electron transfer can be regulated by temperature, pH, and substrate. The reaction also follows the Michaelis–Menten kinetics. Up to date, research on the catalytic mechanism of nanozymes mainly focused on POD-like nanozymes. In contrast, OXD-like nanozymes which work in the absence of H_2_O_2_ are more valuable in practical biological applications. Previous studies revealed that the OXD-like mechanism is similar to oxygen reduction reactions. For example, Cui et al. developed a Fe–N/C–CNT nanomaterial with Fe-N_3_ units through theoretical prediction and experiment, as a typical example of OXD-like nanozymes [120]. The geometrical structure-dependent enzymatic catalytic mechanism of M-N_*x*_ (M = Fe, Co, and Ni;* x* = 0, 3, 4, and 5) models with different configurations was systematically studied and elucidated. They demonstrated that the entire process includes the decomposition of O_2_ and electron-proton pair transfer through both experiments and theoretical calculations, where electrons transfer from TMB to the catalyst to form oxidized state of TMB, accompanied by proton transfer from acidic media. The detailed reaction path mainly consists of several chemical reaction steps of “O_2_ adsorption → O_2_ dissociation → O* atoms → 2O* → 2OH* → H^+^_2_O for desorbing from the catalyst”. Only 0.5 eV absorbed energy requires for releasing the second H_2_O molecule, the other steps are exothermic reactions. The change in reaction energy of O_2_* → 2O* (*E*_*r*_ = *E*_2O*_ − *E*O_2*_) process exhibits a similar trend to the OXD-like activity. Furthermore, it was confirmed that Fe–N/C–CNT nanozymes with Fe-N_3_ units exhibit excellent OXD-like activity.

**Fig. 4:**
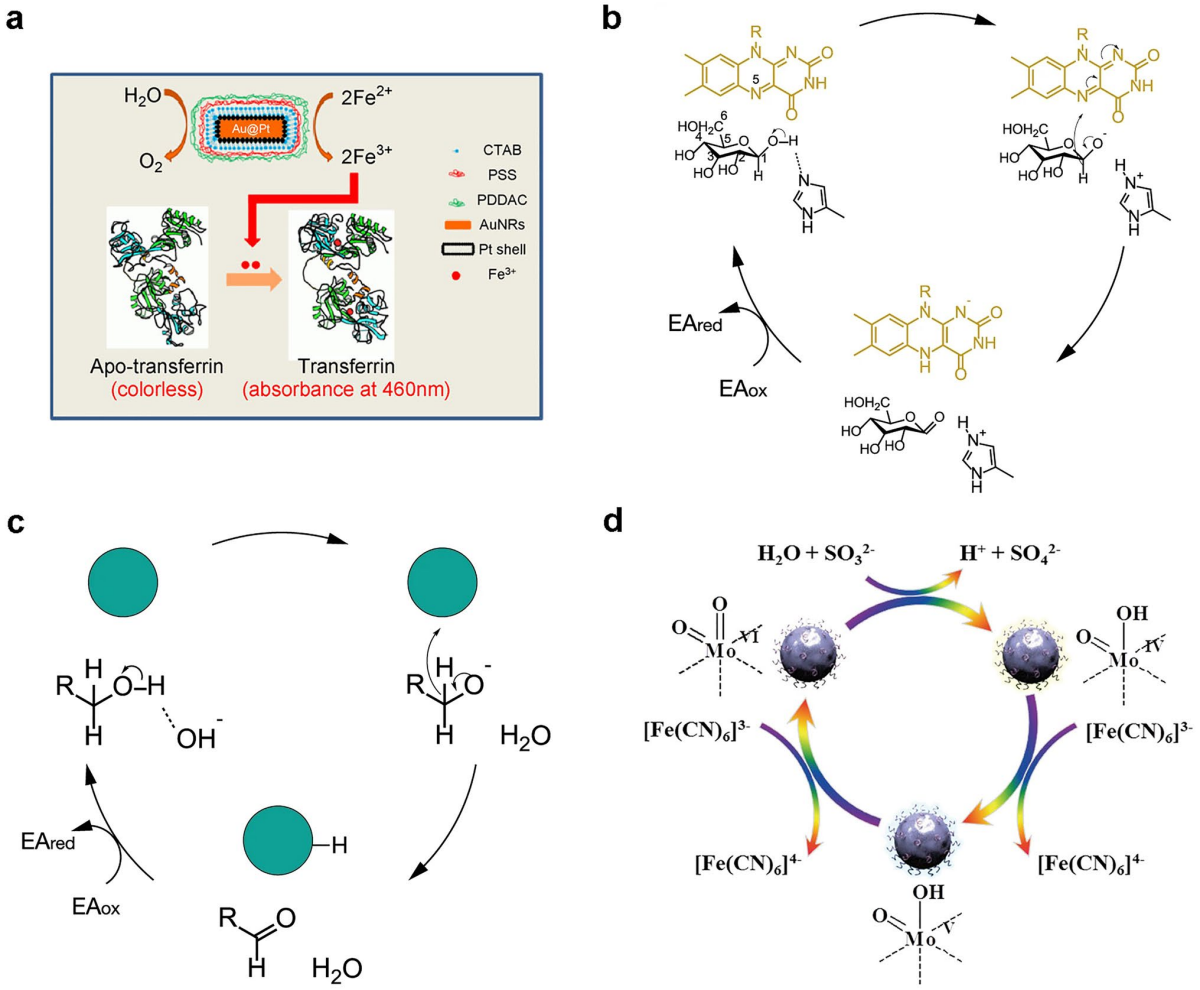
**a** Apo-transferrin determined ferroxidase-like activity of Au@Pt nanorods [121]. Reproduced with permission. Copyright 2015, Tsinghua University Press and Springer-Verlag Berlin Heidelberg. **b**, **c** GOx and noble metal nanoparticles catalyzed glucose oxidation mechanism [122]. Reproduced with permission. Copyright 2021, Springer Nature. **d** The catalytic mechanism of MoO_3_ sulfite-oxidase activity [123]. Reproduced with permission. Copyright 2019, WILEY–VCH Verlag GmbH & Co. KGaA, Weinheim

As a member of OXD-like enzymes, ferroxidase plays an important role in tuning iron homeostasis during life processes. In 2011, Wu et al. discovered that Au@Pt nanorods can catalyze the conversion of Fe(II) to Fe(III) in the presence of dissolved O_2_ (Fig. 4a) [121]. The results showed that compared to natural ferroxidase, Au@Pt nanorods possess higher activity, although their affinity for Fe^2+^ is much lower than that of natural ferroxidase. The simulated activity of ferroxidase in Au@Pt nanorods is pH dependent, typically, with higher catalytic activity at neutral pH values. Therefore, cells can be protected from oxidative stress, because the Au@Pt nanorods can consume excessive Fe^2+^ in the physiological environment. In addition, PtCu nanoparticles also possess strong ferroxidase-like activity in weak acidic environments.

Glucose is the substrate of the most reported OXD-like nanozyme. Natural GOx has the ability to catalyze *β*-D-glucose to generate gluconic acid, and reduce O_2_ to H_2_O_2_. In 2002, Rossi and co-workers studied the effect of carbon-supported gold catalysts on the selective oxidation of *β*-D-glucose [124]. Subsequently, they found that bare Au nanoparticles also catalyzed O_2_ and oxidated *β*-D-glucose, when the Au hydrosol was not protected by carriers or protective agents [125]. Dong et al. compared the GOx-like catalytic mechanisms of natural GOx and noble metal nanozymes [122]. For natural GOx, glucose is first activated by the blocked His residue as Brønsted base in order to remove C1 hydroxyl protons and form intermediates (Fig. 4b). The removal of glucose hydroxyl protons promotes the transfer of hydrides from C1 of glucose to the isoalloxazine ring in flavin adenine dinucleotide (FAD). Then, the C1 position of glucose is directly hydrogenated and transferred to the N5 position in FAD to generate FADH^−^. Then, FADH^−^ can be easily oxidized by O_2_ (electron acceptor, EA) to form H_2_O_2_. The catalytic path of Au nanoparticles is similar to natural GOx, diffferently, OH^−^ is applied as a Brønsted base to extract H^+^ from glucose (Fig. 4c). The Brønsted base of OH^−^ is favorable for the fast oxidation of glucose and alcohol under alkaline conditions. As reported, theoretical calculations show that the decisive step for the oxidation rate of alcohols is to destroy the C − H bond. Notably, Au exhibited the highest catalytic performance due to the strongest affinity to reduce the breaking energy of the C − H bond. For the oxygen reduction reaction catalyzed by Au, high energy barrier requies to break the O = O double bond, thus, mainly 2e^−^ path adopts to produce H_2_O_2_.

Sulfite oxidase is a natural Mo-dependent CAT from the metabolism and decomposition processes of cysteine, which can convert toxic sulfites into sulfates. In 2014, Tremel et al*.* first discovered MoO_3_ nanoparticles with sulfite oxidase-like activity, which can be used to treat sulfite oxidase deficiency [126]. The possible catalytic mechanism is attributed to the bonded sulfite on MoO_3_ nanoparticles. Then, two-electron oxidation hydroxylation follows to produce sulfate, accompanied by reduction from Mo(VI) to Mo(IV) (Fig. 4d) [123]. As reported, bulk MoO_3_ exhibits low sulfite oxidase-like activity. Surface defect engineering is considered as a way to boost the catalytic activity. For example, Yang et al. designed a PEG-based MoO_3−x_ nanoparticles with massive oxygen vacancies, which exhibited a 12-fold increase in sulfite oxidase-like activity compared to those without oxygen vacancies [123]. It is worth noting that monolayer MoO_x_ nanosheets assisted with local surface plasmon resonance also hold efficient sulfite oxidase-like activity, and their catalytic activity is improved by the photothermal effects and generation of hot carriers [127].

### CAT-like Nanozymes

Among numerous redox-like nanozymes, CAT attracted the attention of many researchers due to its ability to catalyze the decomposition of H_2_O_2_ into O_2_ and H_2_O molecules, thereby protecting cells from oxidative stress and alleviating hypoxia [128]. The CAT is a representative peroxisome, accounting for about 40% of the total amount of peroxisome enzymes. However, in practical applications, it has non-negligible drawbacks such as inherent poor stability in natural enzymes and difficulty in storage. Nanozymes have also been designed to simulate CAT activity. So far, kinds of materials including metals, metal oxides, metal sulfides, carbon-based nanomaterials, MOFs, and prussian blue have been proven to have CAT-like activity and have been widely used in tumor treatment, immune detection, and other fields [16, 129].

**Fig. 5:**
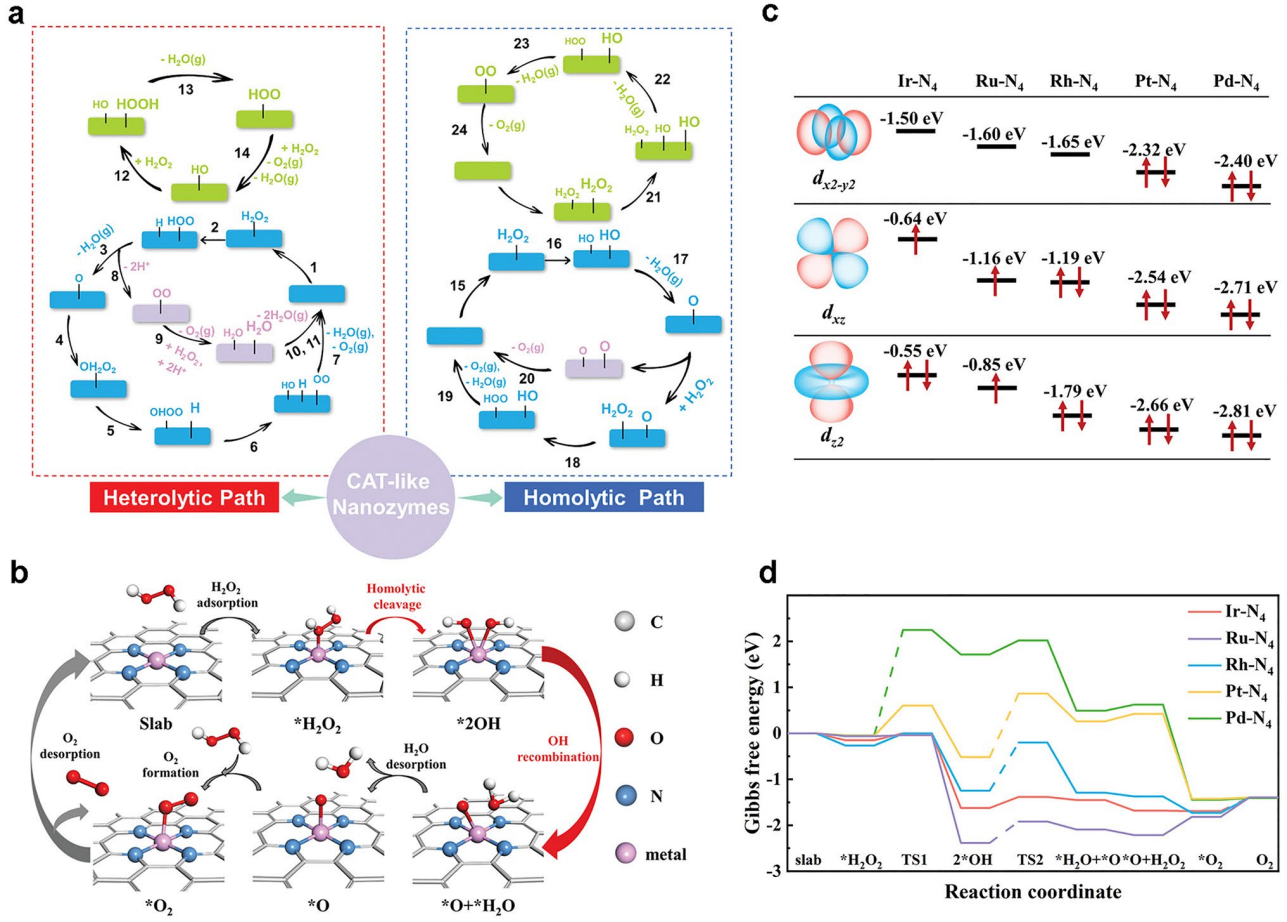
**a** Mechanism diagram of CAT-like nanozymes [129]. Reproduced with permission. Copyright 2022, Wiley–VCH GmbH. **b** Reaction pathway of H_2_O_2_ decomposition on single-atom enzyme mimics. **c** The orbital filling and energy distribution of *d*_x2−y2_ and *d*_z2_ orbitals of metal active centers in M-N_4_ (M = Rh, Ir, Ru, Pt, and Pd). **d** Gibbs free energy diagram of H_2_O_2_ decomposition process on the surface of M-N_4_ model [130]. Reproduced with permission. Copyright 2022, Wiley–VCH GmbH

Exploring the intrinsic catalytic mechanism is important for the advancement of CAT-like nanozymes. Based on DFT calculations, a range of theoretical studies to reveal the catalytic mechanism have been carried out, which proved that CAT-like catalytic mechanisms are different. Generally speaking, H_2_O_2_ has two types of chemical bonds, namely H − O bond and O − O bond. According to the bond cleavage modes of H_2_O_2_, the catalytic mechanisms of CAT-like nanozymes can be summarized into two categories of heterolytic cleavage mechanism and homolytic catalytic mechanism (Fig. 5a) [129]. Among them, the heterolytic cleavage mechanism indicates that H_2_O_2_ molecules are more inclined to break the H − O bond to generate proton and carboxyl-adsorbed species. On the contrary, the homolytic catalytic mechanism indicates that H_2_O_2_ will firstly break the O − O bond and evenly decompose into two hydroxyl-adsorbed species. Accordingly, nanomaterials with suitable oxygen vacancies, pH values, and morphological properties are more likely to have good CAT-like activity by optimizing electronic properties. The above rules can also be applied to CAT-like nanozymes of MOFs and metal sulfides.

In recent years, single-atom catalysts have become a hot research topic in the field of catalysis. Single-atom catalysts refer to a special type of supported metal catalysts formed by the stable formation of metal active components on a carrier as isolated atoms, which show significantly different activity, selectivity, and stability from traditional nanomaterials due to the unique electronic and geometric structure. In 2011, Zhang et al. put forward the new concept of “single-atom catalysts” [131]. The number of metal atoms in the active site of single-atom catalyst can be one or more. Each active site has the same structure, and the interaction between each site and the reactant is also the same. The active site of a single-atom catalyst is generally composed of a single metal atom and other adjacent atoms on the surface of the support (usually forming a certain coordination structure), and the catalytic activity is usually determined by this coordination structure. Single-atom catalysts not only have extremely low metal loading amount, but also greatly improve the utilization rate of metal atoms. Thus, the reaction kinetics are affected due to the change of adsorption/desorption selectivity of the active components on the catalyst towards different molecules.

Thus, single-atom catalysts are ideal candidates for promoting H_2_O_2_ decomposition performance and understanding of H_2_O_2_ decomposition mechanisms. Wu and co-workers reported a strategy for regulating the CAT-like activity of single-atom catalysts through electron filling and orbital energy (Fig. 5b-d) [130]. First, theoretical calculations revealed that the electronic distribution and orbital energy of metal active centers affect the interaction between active centers and H_2_O_2_, thereby affecting the decomposition activity of H_2_O_2_. The metal active center contains unoccupied or partially occupied *d*_x2−y2_, *d*_z2_, and *d*_xz_ orbitals with high orbital energy, which can enhance the interaction between the metal active center and H_2_O_2_, thus, reducing the energy barrier for H_2_O_2_ decomposition and exhibiting higher H_2_O_2_ decomposition activity. The Ir–N_4_ catalytic site, which partially occupies the *d*_x2−y2_ and *d*_xz_ orbitals and has the highest *d*-band center, can effectively interact with the substrate H_2_O_2_ to exhibit the highest CAT-like activity. The results demonstrated that the iridium-based single-atom catalyst (Ir-NC) with Ir-N_4_ active center possesses ultra-high H_2_O_2_ decomposition ability under neutral conditions, typically, 1614 times higher than that of natural CAT. The observed surface-adsorbed atomic oxygen (O*) is a key intermediate for the generation of O_2_. Based on the high H_2_O_2_ decomposition activity and good biocompatibility, Ir-NC can effectively eliminate ROS produced within cells under oxidative stress conditions, protect biological macromolecules such as DNA within cells, and thereby improve cell survival under external oxidative stress. Accordingly, Ir-NC has good potential in clinical treatment related to oxidative stress.

### SOD-like Nanozymes

The SOD is a group of metalloenzymes, which has the ability to catalyze the disproportionation of O_2_^·^^−^ to produce H_2_O_2_ and O_2_ [132]. Natural SOD is usually composed of protein and metal cofactors, which is important for aerobic cells. Therefore, natural SOD widely exists in prokaryotic cells, eukaryotic cells, and organelles. Based on the difference of cofactor, natural SOD enzymes normally can be classified into four categories, which are manganese-based SOD, iron-based SOD, copper/zinc-based SOD, and nickel-based SOD [133]. To prevent ROS-mediated damage, SOD exhibits significant therapeutic effect on oxidative stress-related diseases. Unfortunately, the high cost and structural instability of SOD limited the practical medical applications. Until 1985, Krato and co-workers discovered that C_60_ possessed O_2_^·^^−^ scavenging activity. This is early evidence for the discovery that nanomaterials could mimic SOD activity, while the definition of nanozymes was not proposed at that time. Thereafter, various nanomaterials with SOD-like performance have been developed gradually. So far, approximately 100 types of SOD-like nanozymes have been reported, among them, fullerene and CeO_2_ are the most well-known [134, 135].

**Fig. 6:**
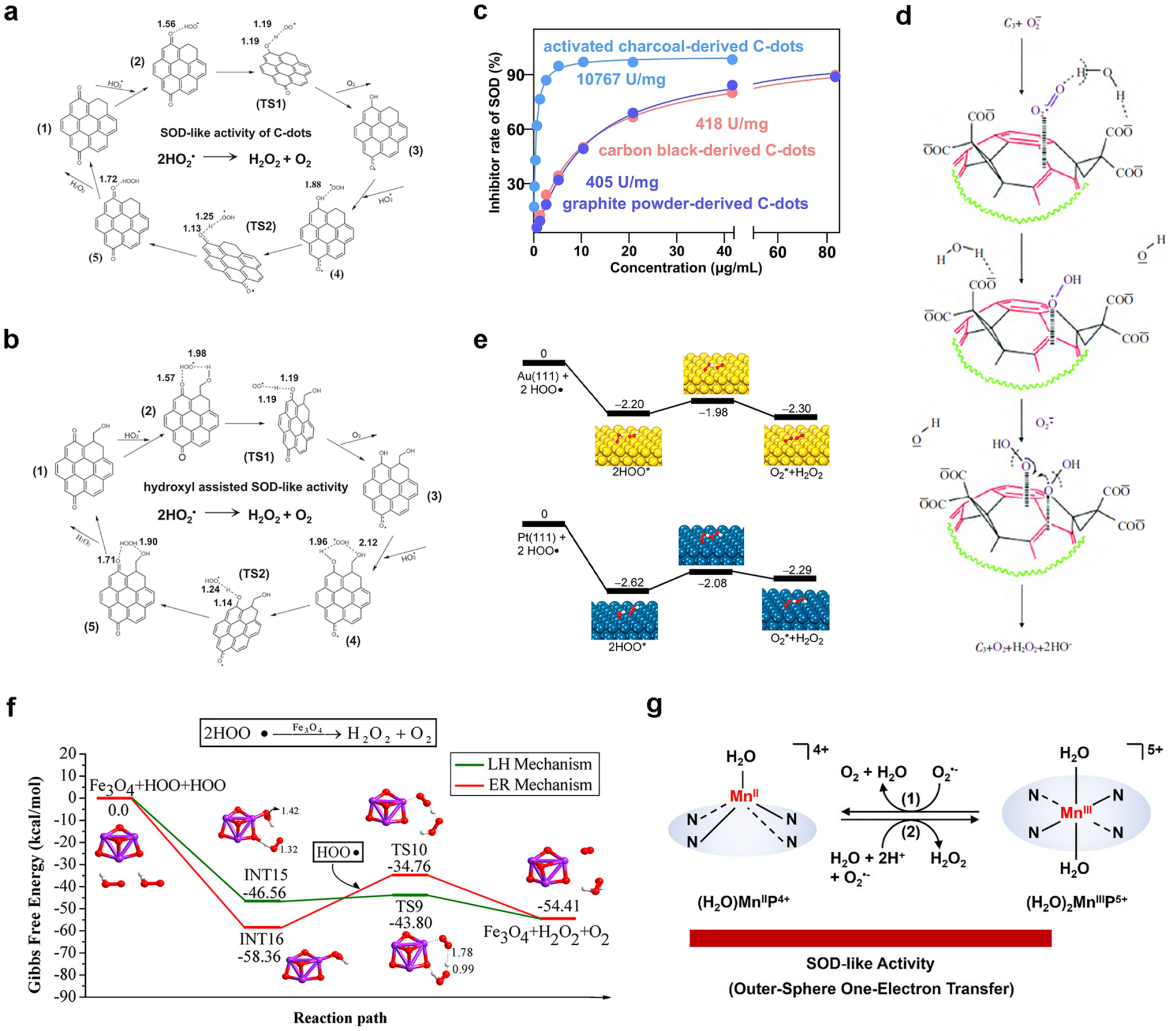
**a, b** Schematic reaction pathway of C-dot nanozyme without and with hydroxyl groups. **c** The SOD-like activities of C-dots [65]. Reproduced with permission. Copyright 2023, Springer Nature. **d** Proposed diagram of the catalytic interaction between C_3_ and O_2_^·^^−^ [136]. Reproduced with permission. Copyright 2004, Elsevier Inc. **e** Rearranging the potential energy distribution of HO_2_· on the (111) surface of gold and platinum [137]. Reproduced with permission. Copyright 2015, American Chemical Society. **f** Calculation the reaction energy curve corresponding to the SOD-like activity of Fe_3_O_4_ [138]. Reproduced with permission. Copyright 2019, American Chemical Society. **g** Chemical mechanism for SOD-like activities of MnTE-2-PyP^5+^ [139]. Reproduced with permission. Copyright 2021, American Chemical Society

In 2023, Yan et al. revealed the SOD-like activity mechanism of C-dot, as shown in Fig. 6a, b [65]. With the assistance of DFT calculation, the proposed reaction pathways are indicated in the following formula.

1-1 $${\text{O}}_{2} ^{\cdot - } \; + \;{\text{H}}_{2} {\text{O}}\; \to \;{\text{HO}}_{2} \cdot \; + \;{\text{OH}}^{ - }$$

1-2 $${\text{2HO}}_{2}^{\cdot} \to {\text{O}}_{2} \;+\; {\text{H}}_{2} {\text{O}}_{2}$$

1-3 $${\text{HO}}_{2}^{\cdot} \; + \;{\text{O}}\; = \;\left( {{\text{C}} - {\text{dot}}} \right)\; = \;{\text{O}}\; \to \;{\text{O}}_{2} \; + \;{\text{HO}} - \left( {{\text{C}} - {\text{dot}}} \right) - {\text{O}}$$

1-4 $${\text{HO}}_{2}^{\cdot} \;+\; {\text{HO}} - \left( {{\text{C}} - {\text{dot}}} \right) - {\text{O}}\cdot \to {\text{H}}_{2} {\text{O}}_{2} + {\text{O}} = \left( {{\text{C}} - {\text{dot}}} \right) = {\text{O}}$$

Clearly, carbonyl groups are the catalytic sites for SOD-like activity, and hydroxyl groups are the key structures. In Fig. 6c, a commercial SOD assay kit was used to quantify the SOD-like activity. The C-dots synthesized from activated charcoal possessed the highest activity about 11,000 U/g owing to the unique structural features of raw materials.

Dugan et al. found that the SOD-like activity of fullerene can be explained as O_2_^·^^−^ disproportionation, instead of simple stoichiometric clearance. There are electron defects on the surface of C_60_–C_3_, which enhance O_2_^·^^−^ adsorption and disproportionation. They also revealed that the SOD-like activity will be increased by the molecular symmetry, polarity, and number of carboxyl groups on fullerene. The unique catalytic mechanism is shown in Fig. 6d [136]. Moreover, some metals, such as Au, Mn, Pt, Fe, and V, as well as their oxides, nitrides, sulfides, and carbides, also possess SOD-like activity. The catalytic mechanism is related to O_2_^·^^−^ protonation, adsorption, and rearrangement of HO_2_· on metal surface, because the HO_2_· adsorption process on the surfaces of gold, silver, palladium, and platinum is highly exothermic [137]. As shown in Fig. 6e, the rearrangement of HO_2_· on Au and Pt surfaces has a very low potential energy distribution, namely, as soon as the HO_2_· is adsorbed on the surface, it will be converted into adsorbed O_2_^*^ and adsorbed H_2_O_2_^*^ easily. Afterwards, O_2_^*^ and H_2_O_2_^*^ desorb into O_2_ and H_2_O_2_.

Currently, CeO_2_ nanoparticles have been widely recognized as candidates for SOD-like nanozymes. Unlike other trivalent lanthanide elements, cerium exists in the form of Ce^3+^ or Ce^4+^. In addition, due to the presence of oxygen vacancies, CeO_2_ nanoparticles have been proven to have excellent catalytic performance. Ghibelli and colleagues proposed a detailed catalytic mechanism for the SOD-like activity of CeO_2_ nanoparticles [140]. The initial state of CeO_2_ nanoparticles is marked as ④, and O_2_^·^^−^ will preferentially combine with the oxygen vacancies around two Ce^3+^ ions to form state ⑤. Then an electron will transfer from a Ce^3+^ ion to an oxygen atom, forming an electronegativity oxygen atom. Afterwards, the obtained two negatively charged oxygen atoms further combine with two protons to form a H_2_O_2_ molecule and intermediate state ⑥. The residual oxygen vacancies bind to another O_2_^·^^−^, resulting intermediate formation ⑦. After that, with the conversion of Ce^3+^ to Ce^4+^, another molecule H_2_O_2_ and state ① are generated. The oxygen vacancies on the surface ① can guide Ce^4+^ to bind the H_2_O_2_ molecule, which results in the intermediate state ②. Intermediate state ② is unstable and will release two protons, accompanied by the conversion of Ce^4+^ to Ce^3+^ to form intermediate state ③. At last, oxygen will be released by converting state ③ to the initial state ④. Therefore, the Ce^3+^/Ce^4+^ ratio on the surface of CeO_2_ has a significant influence on catalytic performance, typically, a higher ratio can enhance the SOD-like activity. In addition, Guo and co-workers evaluated whether the SOD-like mechanism of a series of nanomaterials is similar to Langmuir–Hinshelwood (LH) or Eley–Rideal (ER) processes by using DFT and microkinetic study (Fig. 6f) [138]. Taking (Fe_3_O_4_)_*n*_ (*n* = 1 ~ 2) as an example, several intermediate transition states in LH and ER routes were listed, and the energy distribution of the reaction in each transition state was calculated. Then, the exothermic performance on the potential energy surface was evaluated, which demonstrated that both Fe_3_O_4_ and (Fe_3_O_4_)_2_ reacted by using the LH pathway.

Besides, Shi and co-workers designed a natural anti-oxidase mimic by loading cationic porphyrin ligands into a Mn-engineered mesoporous silica (MnTE-2-PyP^5+^) [139]. In a mildly acidic environment, manganese ions and porphyrin ligands in the mimic will be released, which coordinate and synthesize manganese porphyrins under the coordination environment, exhibiting active manganese sites similar to the metal sites in natural SOD and CAT. Further studies showed that because of the strong metal–ligand exchange coupling of the meta tetrasubstituted N-ethylpyridinium-2-yl groups of the N_4_-macroheterocycles, MnTE-2-PyP^5+^ showed SOD-like activity of disproportionated superoxide anion through the outer proton-coupled single electron transfer, namely, diaquamanganese(III)/monoaquamanganese(II) cycling in Fig. 6g.

### Hydrolytic Nanozymes

Hydrolase is a special class of transferases that can catalyze hydrolysis reactions by using water as a receptor for the transferred group. Lots of unique chemical bonds can be hydrolyzed by hydrolase. For example, nuclease has the ability to cleave the phosphodiester bond of the polynucleotide chain, and esterase can hydrolyze the ester into acid and alcohol. Hydrolytic nanozyme is a nanomaterial with the activity similar to hydrolase, which can be divided into phosphatase, nuclease, protease, esterase, etc*.*, based on the kinds of hydrolytic substrates [141, 142]. Recently, numbers of hydrolytic nanozymes have been reported, for example, nanoparticles, vesicles, and micelles [143–145]. Compared with micelles and vesicles, the topological structure and catalytic sites of nanoparticles can be well controlled. Thereafter, nanoparticle-based hydrolytic nanozymes are well reported.

**Fig. 7:**
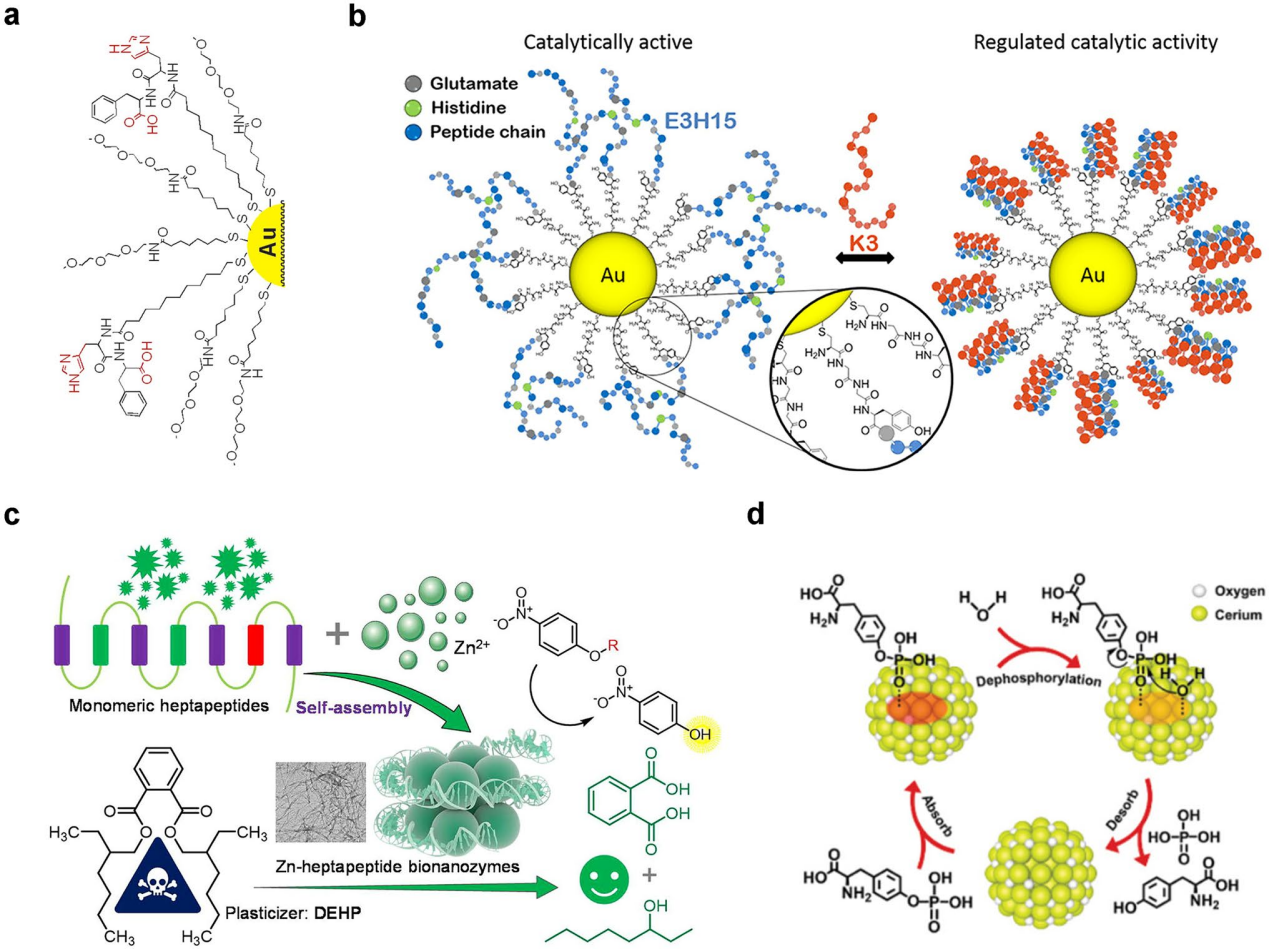
**a** Phenylalanine/histidine dipeptide on Au nanoparticles [148]. Reproduced with permission. Copyright 2005, American Chemical Society. **b** Conformational change of self-assembled peptide monolayer [150]. Reproduced with permission. Copyright 2017, American Chemical Society. **c** Schematic illustration showing the construction of zinc heptapeptide self-assembled bionanozyme, the hydrolase-like activity and DEHP degradation [151]. Reproduced with permission. Copyright 2022, Elsevier Inc. **d** Phosphatase-mimetic activity of PMNSs [152]. Reproduced with permission. Copyright 2021, Wiley–VCH GmbH

As we know, surface modification of Au nanoparticles with thio-ligands to form organic monolayers is easy [146, 147]. The organic monolayers are very important because the catalytic sites are generated in them, instead of Au nanoparticles. Thus, the activity of hydrolytic nanozymes can be controlled by changing or assembling the organic monolayers. For example, imidazole groups are important for the hydrolysis of biological substances. Therefore, imidazole groups, imidazole complexes, and histidine-containing peptides have been assembled onto Au nanoparticles to design hydrolytic nanozymes. Pasquato and co-workers studied the hydrolysis of 2,4-dinitrophenyl acetate catalyzed by N-methylimidazole-modified Au nanoparticles [144]. Compared with the acetyl-*N*-methylhistamine monomer, the catalytic efficiency of monomer-modified Au can be increased to 30 times under the same conditions. In addition, when peptides were self-assembled on the surface of Au nanoparticles, effective nanozymes could be developed. Scrimin et al. modified dipeptides on Au nanoparticle surfaces with phenylalanine carboxyl and histidine imidazole groups (Fig. 7a) [148]. By taking advantage of the synergistic effect of both groups, the hydrolysis rate can be enhanced nearly 300 times. However, dipeptide monomers do not possess this synergistic effect, and only imidazole participates in the hydrolysis reaction, resulting in low activity. Moreover, the high-density dipeptides on the surface of Au nanoparticles produce a microenvironment, which is similar to the active site in natural enzymes. In addition, changes in the monolayer structure of self-assembled peptides interfere the nanozyme activity [149]. Koksch et al. designed another hydrolytic enzyme activity regulation method for peptide-nanoparticle conjugates by tuning the peptide conformation from random curl to coiled helical structure. As shown in Fig. 7b, E3H15 peptide is covalently linked to Au nanoparticles through Au–S bonds [150]. In comparison with non-immobilized E3H15, E3H15-immobilized Au nanoparticles could trigger the cooperative glutamate-histidine interaction, and the hydrolysis efficiency of 4-nitrophenyl acetate (4-NPA) could increase more than one order.

Inspired by the basic unit of histidine in the active center of natural hydrolase and the structure of zinc ion cofactor, Lou et al. constructed a zinc heptapeptide self-assembled hydrolase-like bionanozyme with divalent zinc and three histidine-rich heptapeptides as the basic structural unit (Fig. 7c) [151]. The biological nanozymes possess *β*-sheet secondary conformation and ordered nanofiber-like morphology, which can catalyze the hydrolysis of various *p*-nitrophenyl ester model substrates. The application in degradation of plasticizer di(2-ethylhexyl) phthalate (DEHP) was explored, providing theoretical reference and practical example for guiding the development and application of biological nanozyme. Wei et al. prepared a ceria-based phosphatase-mimetic nano-stabilizers (PMNSs) [152]. As shown in Fig. 7d, after the dephosphorylation reaction, the P–O stretching vibration disappers, demonstrating that both the Tyr and free phosphate are desorbed, which helps to regenerate the catalytic hotspots for the next round.

Furthermore, the catalytic activity of hydrolytic nanozymes can also be influenced by changing the position of catalytic sites in peptide monolayer. Koksch et al. reported the moved histidine along the heptad repeat region sequentially to obtain E3H15, E3H8, and E3H22 peptides. Then, they were coupled onto the surface of Au nanoparticles to obtain three types of peptide-Au nanozymes with different catalytic sites (histidine) in the peptide chain. The results showed that the ester hydrolysis rate was influenced by substrate hydrophobicity and catalytic site position. That is, substrates with lower hydrophobicity exhibit higher hydrolysis rates in the intermediate region of the organic monolayer [153]. In peptide-Au nanozymes, Au nanoparticles catalyze the occurrence of hydrogenation or oxidation to achieve a cascade process. On this basis, Koksch and co-workers modified Au nanoparticle surface by using E1H8 peptide with 11 amino acids to form AuNP@E1H8. The designed AuNP@E1H8 combines both the hydrogenation and esterase activities to catalyze two different chemical reactions in a controllable manner under the same conditions. Acting as an esterase, the self-assembled peptide monolayer catalyzes the hydrolysis of 4-NPA to produce 4-nitrophenol. Then, Au nanoparticles may catalyze the reduction of 4-nitrophenol to generate 4-aminophenol [154].

Wei et al. analyzed 105 published papers on hydrolytic enzymes systematically through data screening to explore high-performance hydrolytic nanozymes [155]. Data analysis shows that MOFs are an ideal scaffold for embedding adjustable metal clusters to hydrolyze active site and ligands. More structure data show that there are two key factors, one is the Lewis acidity of metal clusters in MOFs, and the other is the density of active site regulated by ligands. Therefore, Ce^4+^ was screened as a promising metal ion, then, short ligand fumaric acid (FMA) was combined to construct homologue UiO-66 (Ce–FMA). First, even without the use of a co-catalyst, the inactive Ce-FMA exhibits good phosphatase-like activity. Second, Ce–FMA has high bovine serum albumin hydrolysis activity, with an efficiency about 12.7 times than that of Zr-based MOF-808. Finally, Ce–FMA was applied to the mixture of biomacromolecules from gram-negative bacteria (*E. coli*) and gram-positive bacteria (*S. aureus*), which confirmed that the decrease of biofilm formation was due to hydrolysis instead of bacterial cell death.

## Activity Prediction and Rational Design of Nanozymes

Due to the complex components and fuzzy catalytic sites of nanomaterials, the basic understanding of the relationship between particles and property of nanozymes is lacking. How to efficiently design nanozymes is one of the key issues that urgently need to be solved in the field. At present, experience and “trial and error” methods are commonly used in the design and synthesis of nanozymes. The preparation of nanozymes with desired features usually requires repeated experiments and is based on research’s intuition and experience. These methods are labor-intensive, time-consuming, and resource intensive. According to the research and statistics on existing POD-like nanozymes, it has been found that the activity of POD-like nanozymes prepared using “trial and error” methods is generally low, typically, several orders of magnitude lower than that of HRP. Therefore, how to efficiently design nanozymes is of great significance for further expanding research and promoting practical applications. Up to date, several strategies for the efficient design of nanozymes were developed, mainly including DFT, machine learning, biomimetic design, and chemical design.

### DFT and Machine Learning

Currently, the first computational revolution in materials science is pushing by the cross-application of materials science and computer science. Traditionally, computational simulation methods mainly include DFT, finite element analysis, molecular dynamics, and Monte Carlo method. By using the theoretical calculations and simulation techniques, researchers can explore more microscopic phase and spatial components effectively, and utilize more abstract energy concepts to handle more complex systems. By combining computational simulation methods to leverage the ability of machine learning, process big data and extend, is a novel research approach in the field of computational materials. Currently, the most widely used material calculation and simulation method is DFT. There are many methods that combine DFT with machine learning. The most direct method is to use the calculation results of DFT as training data for machine learning, and use supervised learning for prediction. Under the ensuring consistency between theoretical and experimental data, machine learning can utilize both experimental and theoretical data for more efficient simulation calculations.

**Fig. 8:**
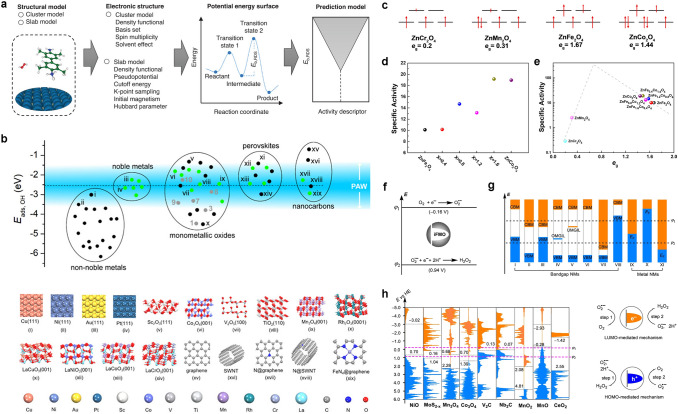
DFT calculation of nanozymes. **a** Procedures of DFT calculations including mechanisms, kinetics, and prediction models of nanozymes [101]. Reproduced with permission. Copyright 2023, Wiley–VCH GmbH. **b** Overview of POD-like activities of nanomaterials and structures of the nanomaterials labelled with Roman numerals [156]. Reproduced with permission. Copyright 2020, American Chemical Society. **c** Electron arrangement of *d* orbitals. **d**, **e** Specific POD-like activities [79]. Reproduced with permission. Copyright 2022, American Chemical Society. **f–h** The energy level principle for nanomaterials to catalytically scavenge O_2_^·−^ [157]. Reproduced with permission. Copyright 2021, Springer Nature

The DFT calculations describe the potential energy surface of reaction system along the reaction coordinate direction at the atomic scale, and reveal the micro-mechanism and kinetics for catalytic reaction. In recent years, DFT played an important role in the research of nanozyme catalytic mechanisms. These calculation results either depict the atomic processes catalyzed by nanozymes, providing support for experimental research, or further develop theoretical models to describe catalytic activity, providing theoretical tools for computational design. Gao and co-workers reviewed the molecular mechanism and basic laws of nanomaterials simulating the catalytic function of biological enzymes [101]. The cross collaboration between computation and experimentation was emphasized. Additionally, procedures of DFT calculations were summarized in Fig. 8a, including several steps of structural model, electronic structure potential energy surface, and prediction model.

Gao et al. also proposed a general method for computer-aided screening and design of POD-like nanozymes, which was used to predict the catalytic activity (Fig. 8b) [156]. DFT method was used to study the mechanisms and kinetics for the POD-like nanozymes. By using the earliest iron oxide nanozymes as models, 15 types of iron oxide surfaces with different chemical compositions, lattice defects, exposed crystal planes, and chemical modifications were constructed. The molecular mechanism and reaction kinetics of these surfaces in simulating POD-catalyzed H_2_O_2_ oxidation of organic substrates were studied, and the three-step mechanism characteristics of this type of catalytic reaction were summarized. Second, based on the principles of chemical reactions, approximate processing, and derivation, further derived energy descriptors, volcano-shaped curves, and catalytic activity windows for predicting the POD-like activity of iron oxide were conducted. Computational validation on 57 metals, single metal oxides, perovskites, and carbon materials was also conducted to prove that the model can be applied to any other POD-like nanozymes with similar catalytic mechanisms. This study confirms that high-throughput computing screening achieves efficient design of nanozymes, accelerates research in the field of nanozymes, and has a certain guiding significance for experiments.

In order to obtain descriptors to guide the design of nanozyme, Wei et al. used perovskite oxide nanomaterials as a model to systematically study the influence of electronic structure of metal oxide on catalytic activity [158]. A clear volcano-shaped relationship between the number of *e*_g_ electrons of transition metal ions in perovskite oxides and their catalytic activity was revealed. When the number of *e*_g_ electrons is around 1, the catalytic activity is highest. When the number of *e*_g_ electrons is 0 or 2, the catalytic activity is the lowest. Therefore, the electron occupation of *e*_g_ can serve as a descriptor for the POD-like activity of perovskite oxide to guide the design and screening of nanozymes. They further elucidated the catalytic mechanism of perovskite oxide nanozymes using theoretical calculations, and theoretically explained why the electron occupation of *e*_g_ can serve as a descriptor for POD-like reactions. The electron occupation of *e*_g_ mainly affects the speed of the entire catalytic reaction by changing the adsorption strength of the material on the substrate and determining the reaction rate step. When the *e*_g_ of transition metal oxides is around 1, they have optimized adsorption energy and can effectively promote the reaction rate of determining step. This conclusion can be further extended to binary metal oxides with BO_6_ coordination. The theory was also used to predict the POD-like activity of Zn-based spinel oxides [79]. As shown in Fig. 8c-e, the final electron arrangement was given, ZnFe_2−x_Co_x_O_4_ series and Zn-based spinel oxides all possessed good POD-like activity. Both reports not only break through the limitations of trial-and-error strategies, but also propose the activity descriptor of POD-like enzymes for the first time. More importantly, they will inspire researchers to rationally design efficient nanozymes with other enzyme-like activities.

Compared to SOD, nanomaterials are more stable and cheaper. However, the activity in the catalytic elimination of superoxide anion is similar to that of SOD. Although several tens of nanomaterials have been proven to have such activity, their fundamental principles are still unclear, which hindered the discovery of new SOD-mimicking nanomaterials. DFT was used to reveal the thermodynamics and kinetics of the catalytic process by Gao and co-workers [157]. The energy level principle and the adsorption energy principle are developed for activity calculation. The former reveals the influence of intermediate frontier molecular orbital in electron transfer on catalysis quantitatively (Fig. 8f-h). The second quantitative description indicates the competition between desired catalytic reactions and unwanted side reactions. Experiments prove that the principle is able to predict the SOD-like activity of MOFs. Both of them can be implemented in computer programs easily to calculate and screen SOD-like nanomaterials.

The excellent properties of nanomaterials broadened the means for regulating nanozyme activity. With the continuous development of synthesis technology, the number of nanozymes prepared in experiments is also increasing. However, how to efficiently obtain high-performance nanozymes from such a vast material library is becoming a huge challenge. In recent years, computer-assisted high-throughput screening has become a popular and effective screening method, which can quickly evaluate a specific performance from a large number of materials and obtain the required materials. As a branch of artificial intelligence, machine learning is brought with the purpose of developing computational algorithms and infusing mathematical models based on existing data, providing a promising tool for designing required nanoparticles [159, 160]. Taking into account the complexity and diversity of nanozymes, researchers consider whether machine learning can reveal the relationship between the structure and catalytic performance for efficient design.

**Fig. 9:**
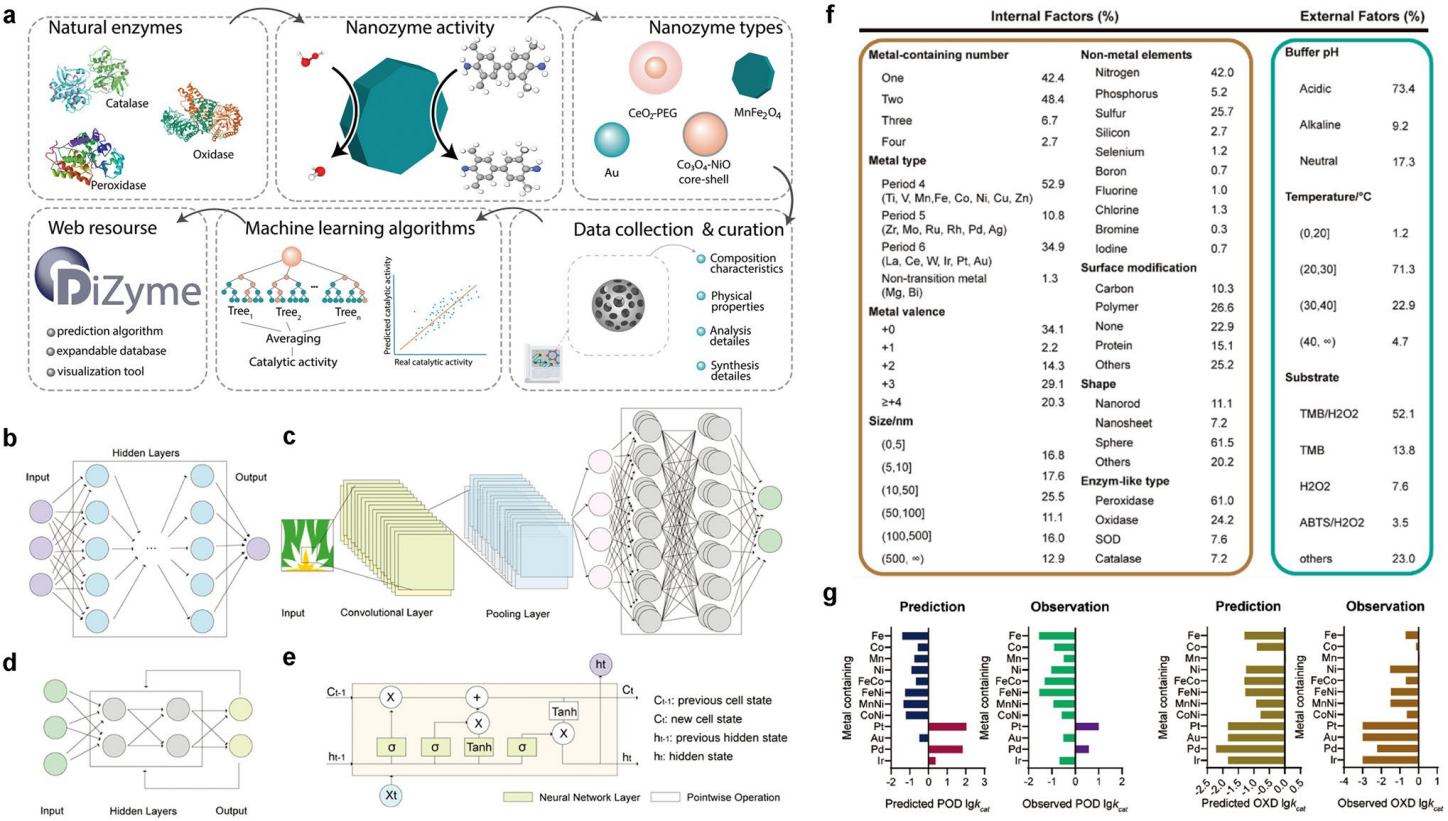
Machine learning process of nanozymes. **a** Resource development schematic diagram from collecting database to web resource DiZyme [161]. Reproduced with permission. Copyright 2022, Wiley–VCH GmbH. Scheme of different artificial neural networks. **b** ANN. **c** CNN. **d** RNN. **e** LSTM [162]. Reproduced with permission. Copyright 2023, Wiley–VCH GmbH.** f** Distribution of the internal factors and external factors from 920 pieces of data. **g** Experimental validation of the predicted POD-like and OXD-like activity from quantitative model [159]. Reproduced with permission. Copyright 2022, Wiley–VCH GmbH

Vinogradov et al. designed the basic idea of using machine learning to develop and predict nanozymes, including analysis and classification of natural enzyme activity, data collection and curation, machine learning algorithms, and web resources of prediction algorithm, expandable database, and visualization tool, as shown in Fig. 9a [161]. Data collection from more than 100 articles was analyzed to reveal the catalytic features of nanozymes. A random forest regression model was optimized to evaluate POD-like activity, namely, the activity determined by coefficient of determination (*R*^2^) was up to 0.796 for catalytic rate constant and *R*^2^ = 0.627 for *K*_m_. Huang and co-workers summarized the machine-learning-assisted catalytic materials and nanozyme design [162]. They focused on machine learning algorithms and database, including the basic principle of machine learning, types of machine learning models, material database, and enzymatic engineering database. Taking artificial neural networks as an example, all types of all-connected neural network (ANN), convolutional neural network (CNN), recurrent neural network (RNN), and long short-term memory (LSTM) are schemed in Fig. 9b-e.

Huang and co-workers also proposed a data-driven method to reveal the particle-property relationships by utilizing machine learning algorithms, which achieved nanozyme classification and quantitative prediction of activity [159]. In order to establish a nanozyme database, over 300 papers were reviewed to extract data from the intrinsic enzyme-like activity of nanomaterials. After the database was established, two machine learning models were constructed to predict different objectives. The first one was enzyme-mimicking type, which is also named as classification model. The other one was the level of enzyme-like activity, also named as quantitative model. They chose fully connected models based on deep neural networks (DNNs) because they can handle unstructured, unlabeled, and nonlinear data. Two types of internal and external factors as extracted data were collected (Fig. 9f). The factors are first filled into the input module as independent variables. After that, the enzyme-mimicking type (i.e. dependent variable) is output by applying the classification model. For quantitative models, the activity level of each type of enzyme is set as the dependent variable by standardizing the enzyme activity coefficient. In addition, they also set the regularization of the model and validation data to overcome the potential over fitting of the model. By analyzing data on established models, the authors accurately predicted high POD- and OXD-like activities of Ru-based and Mn-based nanomaterials respectively, both of which were validated in the experiment (Fig. 9g).

Accordingly, the following steps can be used for prediction of enzyme activity in nanomaterials. The first step is data collection. All kinds of parameters from large amounts of nanozymes are collected from the reported articles and computer simulations, including the intrinsic material parameters (e.g., elemental composition, crystal structure, energy band, formation energy, electronic structure, particle size, morphology), functional parameters (e.g., catalytic activity and type), and synthesis parameters (e.g., synthesis method and condition, surface modification). The second step is feature engineering. A nanozyme database for machine learning is established by using the computer-based data preprocessing tools, which can perform data cleaning, correlation analysis, outlier handling, missing value handling, and dimensionality reduction. The third step is data guidance. Data analysis, statistical model building and important data summary about nanozymes can be realized by big data analysis. Moreover, taking advantage of model evaluation methods, algorithms, and hyperparameters, the machine-learning models for prediction are evaluated and optimized.

### Biomimetic Design

With the widespread application requirements, the development of nanozymes with catalytic performance comparable to natural enzymes is an important research topic. Reported nanozymes were mostly obtained through random fabrication and screening, while the catalytic activity is difficult to reach the level of natural enzymes. After billions of years’ evolution, natural enzymes evolved many highly efficient catalytic units hidden within their structures. Recently, many reports attempted to graft the core catalytic elements of natural enzymes into the structure of nanozymes through biomimetic simulations. Many factors providing high catalytic activity for natural enzymes have been considered by researchers to guide the structural design of nanozymes, mainly including biomimetic binding sites, defect engineering, composition, and coordination chemistry, thus, a series of nanozymes with high catalytic activity were synthesized. The catalytic activity of some nanozymes from biomimetic methods is close to or even exceeds that of natural enzymes. At present, the design of highly active nanozymes through biologically inspired strategies is becoming a hot research topic [163].

Usually, chemical reactions of substrates can be efficiently catalyzed by natural enzymes with high selectivity and activity. The high catalytic efficiency of natural enzyme can be attributed to the composition and structure of the binding pocket around the active center, which exhibits beneficial hydrophobic microenvironment [164]. Particularly, the substrates can be enriched, organized, and activated by non-covalent interactions from the amino acids located around the catalytic active site of natural enzyme [165, 166]. Encouraged by these specific functions, constructing the catalytic microenvironment near the active site by modifying particular amino acids and building desired spatial structures may be a promising strategy for building nanozymes with catalytic activity closer to natural enzymes. The POD-like activity of Fe_3_O_4_ nanomaterials has been widely reported, because there are massive ferric and divalent irons available on the particle surface, and the catalytic mode is similar to the heme group in the HRP active site. Moreover, the catalytic efficiency (*k*_cat_) of Fe_3_O_4_ is equivalent to that of HRP, while the binding ability and affinity to the substrate are much lower than that of HRP due to the lack of amino acid environment similar to the catalytic center of HRP. For the active site of HRP, both His42 and His170 histidine residues appear. Thus, H_2_O_2_ was assisted by His42 residue to enter the cavity of the active site, since the formed hydrogen bond and the initial compound I help to fix H_2_O_2_ in an appropriate position, promote the heterolysis of the peroxide O − O bond to produce Fe^4+^ = O, and stabilize the high oxidation state of heme iron in the catalytic cycle [78]. Hence, Yan et al. covalently modified histidine residues onto Fe_3_O_4_ surface to form H_2_O_2_ binding sites and enhance the affinity of Fe_3_O_4_ nanozymes to substrates. As expected, the Michaelis–Menten kinetic analysis showed that the affinity of histidine-modified Fe_3_O_4_ (His-Fe_3_O_4_) to H_2_O_2_ was significantly higher than that of unmodified Fe_3_O_4_ (Naked-Fe_3_O_4_), with a tenfold decrease in *K*_m_ value. In addition, the *K*_m_ value of His-Fe_3_O_4_ is significantly lower than that of Fe_3_O_4_ (Ala-Fe_3_O_4_) modified with only methyl alanine, indicating that the enhanced affinity for H_2_O_2_ originates from the imidazole side chain of histidine rather than amino group or carboxyl group. As the affinity increases, His-Fe_3_O_4_ exhibits extremely high catalytic efficiency (*k*_cat_/*K*_m_), namely, 7.1 and 20.8 times higher than Ala-Fe_3_O_4_ and Naked-Fe_3_O_4_ respectively. Mechanism analysis indicates that the formed hydrogen bond between H_2_O_2_ and histidine during the catalytic process weakens the strength of the O − H bond, meanwhile, induces O to carry more negative charges, which are beneficial for the O − O bond cleavage of H_2_O_2_ and the enhancement of H_2_O_2_ adsorption on Fe_3_O_4_ nanozymes (Final state). Therefore, the surface modified histidine plays a similar role to His42 in the active site of HRP, greatly improving the enzymatic activity of Fe_3_O_4_.

**Fig. 10:**
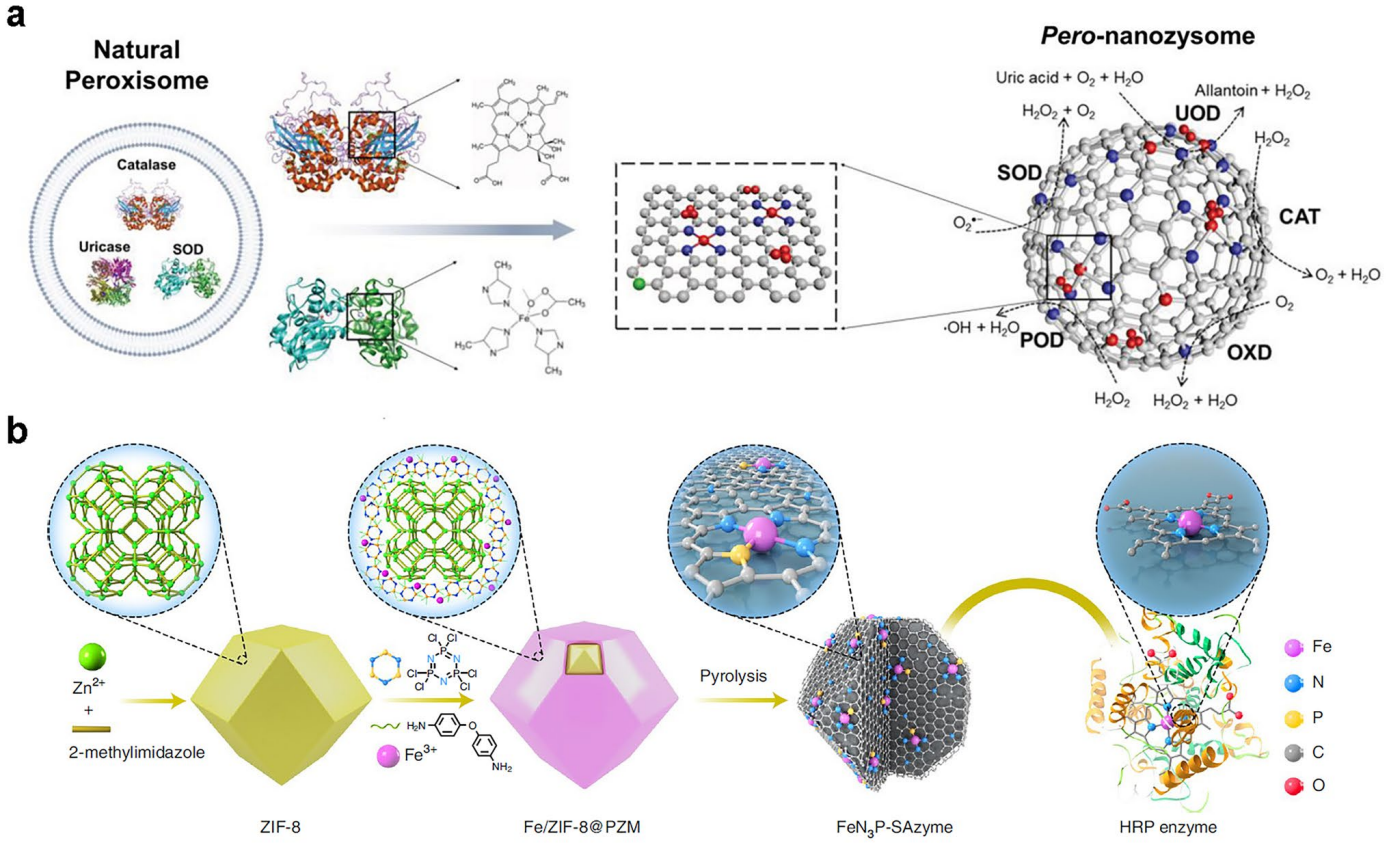
Biomimetic design methods of nanozymes. **a** Schematic diagram of designing artificial peroxisome using iron-doped nanozyme [90]. Reproduced with permission. Copyright 2020, Wiley–VCH GmbH. **b** Synthesis and POD-like activity of FeN_3_P-SAzyme [92]. Reproduced with permission. Copyright 2021, Springer Nature

As active site, metals exist in many nanozymes. Compared with natural enzymes, the metal site amount naked on nanozyme surface is much more, while the catalytic efficiency is lower. Because the availability of metal sites in nanozymes is limited by the random and rough structure, in contrary, the metal ions in natural enzymes are well controlled by coordination structures. In the active center of natural enzymes, metal ion cofactors typically coordinate with donor groups including oxygen, nitrogen, and sulfur that belong to the protein amino acid residues [167]. Besides, in the active site, amino acids further coordinate with metal atoms, and control the orientation of the substrate through non-covalent bond [168]. Furthermore, amino acid residues provide donor groups, and lots of organic cofactors also act as ligands. Among them, the tetradentate N_4_ macrocyclic ligand was embedded in heme protein [169]. Accordingly, as catalytic groups in catalytic reactions and transfer electrons, an optimized coordination structure for metal ion cofactors is crucial. Inspired by this, on the basis of nitrogen-doped carbon nanozyme structure, Yan et al. further introduced Fe element in order to form a Fe − N coordination structure similar to the natural enzyme cofactor, following the structural characteristics and synergy of the active center of natural enzyme and cofactor [90]. Firstly, SiO_2_ synthesized by the Stöber method was used as a template. Then, iron was introduced into the structure of nitrogen-doped carbon to synthesize hollow nanozymes with Fe − N_4_ coordination structure (represented as *pero*-nanozysomes) (Fig. 10a). The structure is very similar to the structural characteristics of iron porphyrins in natural enzyme cofactors. In addition, the nanozyme surface has a structure of Fe clusters formed by iron atoms. The novel nanozyme constructed with cofactors of Fe clusters and prosthetic group of Fe − N_4_ has stable and various enzyme-like activities, such as CAT-, SOD-, POD-, OXD-like, and uric acid oxidase activities. Compared with nitrogen-doped nanozymes, the activity of each type of *pero*-nanozysomes has been significantly improved. Among them, the SOD-like activity reaches 1000 U mg^−1^, and the catalytic activity can be compared with that of natural SOD about 3000 U mg^−1^ from cattle liver. Taking advantage of the cascade reaction between uric acid oxidase/CAT-like and SOD/CAT-like activities, *pero*-nanozyme alleviates hyperuricemia and ischemic stroke in animal models effectively. Moreover, this nanozyme with multi-enzyme activity has many similarities with peroxisome in cells. The *pero*-nanozyme has high stability and several kinds of enzyme activities that are important characteristics in peroxisome, so it is expected to be used as artificial peroxisome for the treatment of related diseases in vivo.

In addition, phosphorus species are crucial in many natural enzymes for assisting electron shuttles from substrates to enzyme active centers [170]. On this basis, Yan et al. also developed an engineered FeN_3_P-centred single-atom nanozyme (FeN_3_P SAzyme) to regulate the single-atom Fe active center by precise coordination of P and N (Fig. 10b) [92]. First, zeolite imidazole framework-8 (ZIF-8) was prepared as carbon and nitrogen precursor through the solvothermal method. Then, Fe ions were polymerized and coated on the surface of ZIF-8 using the precursor poly-(cyclotriphospazeneco-4,4′-diaminodiphenylether) (PZM) monomer of Fe and P to obtain Fe/ZIF-8@PZM core–shell composites. After high-temperature pyrolysis, FeN_3_P-SAzyme was obtained. Compared with HRP, the synthesized FeN_3_P-SAzyme has a similar chemical coordination environment, which can catalyze the oxidation and color reaction of HRP substrates effectively, such as diazoaminobenzene, TMB, and o-phenylenediamine in the presence of H_2_O_2_. Compared to the most widely used Fe_3_O_4_ nanozymes and FeN_4_-SAzyme with catalytic activities of 9.12 and 33.8 U mg^−1^ respectively, FeN_3_P-SAzyme has higher catalytic activity of 316.0 U mg^−1^.

As well known, nature has always been a source of inspiration for creating new chemical, structural, and functional materials. After billions of years, natural enzymes developed many highly efficient catalytic units hidden within their structures. In order to develop highly active nanozymes, researchers assumed a general structure and activity association between natural enzymes and nanozymes, and grafted the catalytic principles of natural enzymes into the design of nanozymes. Under the guidance of the biological system-inspired strategy, researchers have designed and constructed an increasing number of highly active nanozymes by simulating the fine catalytic structure of natural enzymes, the variation of natural enzymes during the catalytic process, and unique biological processes or organisms [163, 171]. For instance, large amounts of studies have focused on designing nanozymes with clear binding structures and catalytic sites to simulate the catalytic microenvironment in natural enzymes, improve their catalytic performance, and synthesize a range of highly efficient nanozymes with catalytic activity. Their catalytic activities are similar to or even exceed that of natural enzymes. In addition, high activity, selectivity, and catalytic specificity nanozymes can be constructed by combing the key catalytic compositions and structures of natural enzymes into nanomaterials. Some special biological behaviors, responses, and organisms, such as the intracellular antioxidant defense system, redox dynamic equilibrium, organelle, microorganisms, and topological interactions in nature, also urge to build nanozymes with high catalytic and therapeutic properties. This biologically inspired perception is expected to arouse worldwide attention in basic research and applications, and provide more inspiration for the reasonable design of nanozymes, enabling precise design and regulation of nanozyme activity.

### Chemical Design

Nanozymes have strong catalytic activity, adjustable specificity, and versatility, which have been expected to be used in biomedical applications. However, the catalytic activity of most existing nanozymes is not satisfactory for applications. Thus, it is crucial to define the correlation between the physicochemical properties and catalytic activity of nanozymes. For the design of nanozyme, idea from the chemical and structural perspectives is a direct way, such as size and facet of nanomaterials, defect engineering, surface ligand engineering (or surface modification), aggregation or dispersion state, and so on [172]. Besides, the deep correlation between the physicochemical properties and catalytic activity of nanozymes can be revealed. It is crucial to gain a deep understanding of the optimization pathways for further improving the catalytic activity of nanozymes.

**Fig. 11:**
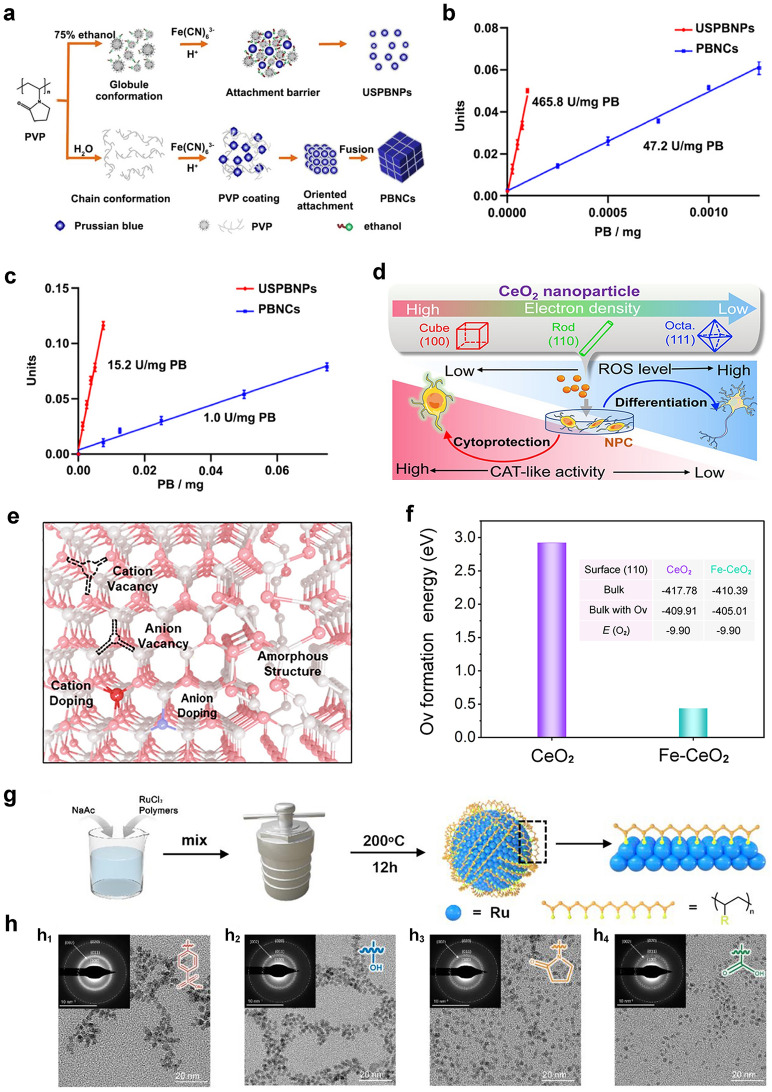
Chemical design methods of nanozymes. **a** Synthetic mechanism of USPBNPs and PBNCs. **b, c** POD- and CAT-like specific activities of USPBNPs and PBNCs [173]. Reproduced with permission. Copyright 2020, American Chemical Society. **d** Mechanism of facet-dependent CAT-like activity tuning of CeO_2_ [174]. Reproduced with permission. Copyright 2022, American Chemical Society. **e** Schematic illustration of different defects in metallic compounds-based nanozymes [175]. Reproduced with permission. Copyright 2021, Elsevier Ltd. **f** Calculated O_v_ formation energies of CeO_2_ and Fe-CeO_2_ [176]. Reproduced with permission. Copyright 2023, American Chemical Society. **g** Synthetic process of Ru-POD nanozymes modified with polymer ligands. **h** Transmission electron microscopy images and corresponding selected area electron diffraction patterns of Ru@PSS, Ru@PVA, Ru@PVP, and Ru@PAA [177]. Reproduced with permission. Copyright 2023, Wiley-VCH GmbH

The size and facet of nanomaterials have significant impact on their properties. Huang et al. explored the effect of nanomaterial size on enzyme catalytic performance by using PBNPs and sub-5 nm ultrasmall PBNPs (USPBNPs) [173]. As shown in Fig. 11a, USPBNPs about 3.4 nm were synthesized by adjusting the ethanol/water ratio, while PBNPs were prepared by conventional method. Because of the large specific surface area, USPBNPs were demonstrated to have higher POD-like and CAT-like activities than that of PBNPs, typically, the specific activities were increased about 10 times (Fig. 11b, c). Besides, both the *V*_max_ and *K*_m_ values of USPBNPs were much larger than those of PBNPs and HRP. On the other hand, Yung et al*.* studied the facet-dependent activity of CeO_2_ nanozymes by using rod-, cube-, and octahedron-shaped CeO_2_ nanozymes [174]. In Fig. 11d, cube-shaped CeO_2_ has the highest CAT-like activity due to the enclosed (100) facet, while octahedron-shaped CeO_2_ has the lowest CAT-like activity due to the enclosed (111) facet. The mechanism can be explained by the influence of the electron density of the surface Ce, which can be changed by different crystal facets. Accordingly, the tuning of size and facet of nanozymes may be a good way to control the catalytic activities.

In the past decade, defect engineering has been widely used for performance tuning of nanomaterials, which has also been used for the control of nanozyme activity. Zhu and co-workers summarized the close relationship between defects and nanozyme properties [175]. Various defect engineering strategies are introduced, including vacancy, functional groups, doping, edge, dislocation, amorphousness, boundary, strain, and cavity (Fig. 11e). Taking the latest research of our group as an example, we designed a mesoporous Fe-doped CeO_2_ (Fe − CeO_v_) nanozymes [176]. Fe-doped was used to realize structural reconstruction and generation of surface oxygen vacancies (O_v_). Then, DFT calculations were carried out to prove that the presence of O_v_ is favorable for catalytic activity. As shown in Fig. 11f, O_v_ formation energy of Fe − CeO_v_ is much lower than that of CeO_v_, demonstrating the easier formation of O_v_ by Fe-doping. Therefore, the *V*_max_ and *K*_m_ values of Fe − CeO_v_ are 7.09 × 10^–8^ M s^–1^ and 22.11 mM, while those values of CeO_v_ are 2.48 × 10^–8^ M s^–1^ and 37.75 mM at room temperature. Namely, both the affinity and catalytic efficiency were enhanced by Fe-doping.

In order to provide additional function or facilitate utilization, nanoparticles typically require surface modification. Thus, the influence of surface modification on nanozyme activity was revealed [178]. In 2023, Gao and co-workers clarified the specific activity of Ru nanozyme influenced by surface ligand engineering [177]. First, Ru nanozymes with different modifications of polystyrene sulfonate (PSS), poly (vinyl alcohol) (PVA), poly (vinyl pyrrolidone) (PVP), and poly (acrylic acid) (PAA) were prepared by solvothermal method (Fig. 11g, h). The results indicate that PSS-modified Ru nanozyme has the highest POD-like specific activity about 2820 U mg^–1^, which is two times higher than that of HRP. To determine the mechanism, DFT calculations were used. The affinity of nanozyme to ·OH was denoted as* E*_ads,·OH_, which was ‒3.68 eV for Ru. By coating PSS and PAA, the charge can transfer from Ru to the coated polymers. The PSS received more negative charge about 0.60 per monomer than that of PAA about 0.27 per monomer. Thus, Ru received fewer electrons, weakened the ·OH affinity, and enhanced POD-like activity. The result was also proved by electron spin resonance (ESR) results. Both DFT calculations and ESR reveal that Ru became less electron-rich after coating with PSS, which weakens the ·OH affinity and enhances the POD-like activity.

Besides, there are other methods for chemical design of nanozymes. Wang et al*.* are interested in the influence of aggregation or dispersion state of MoS_2_ quantum dots [179]. They found that the aggregation of MoS_2_ quantum dots can be achieved by the help of Fe^3+^, and the POD-like activity can also be enhanced greatly. In summary, the chemical design of nanozymes enhances selectivity and activity. Therefore, designing nanozymes with suitable size, high specific surface, customized electronic structures to increase the amount of active sites, etc., are favorable for enhancing the catalytic activity. In addition, although some progress has been made in related research, new methods and in-depth mechanism study still need to be continued to design ideal nanozymes.

In summary, activity prediction and rational design of nanozyme is changing from “trial and error” methods to precise design methods of DFT, machine learning, biomimetic design, and chemical design. For example, DFT is suitable for nanozymes with clear crystal structures, hence, can be used in the design of novel bimetallic nanozymes, metal oxide/sulfide nanozymes, carbon-based nanozymes, MOF-based nanozymes, noble metal nanozymes etc. For biomimetic design, which grafts the catalytic principles of natural enzymes into the rational design of nanozymes, the spatial structure of nanozymes can be customized, thus single-atom nanozymes have been designed, and in the future, may be similar double-atom nanozymes, multi-atom nanozymes and MOF-based nanozymes with different compositions and structures can be designed by this way.

## Synergistic Theranostic Strategy of Nanozymes

Driven by the continuous development of nano-chemistry and nano-catalysis, various nanozymes have been successfully constructed and applied to various biomedical applications [16, 57, 58, 180–184]. In the past decade, the cross-integration of nanotechnology and biomedicine has inspired extensive research on various multifunctional medical nanozymes [67, 185–188]. As well reported, nanozymes with POD-like activity can catalyze the decomposition of H_2_O_2_ and produce highly toxic ·OH to induce apoptosis or necrosis of tumor cells [77, 103, 189–191], which exhibits great potential in catalytic therapy mediated by ROS. However, the complex TME restricts the catalytic activity of nanozymes to some extent, and makes it difficult to realize excellent therapeutic effect [192–195]. Therefore, nanozyme catalytic therapy usually combined with other therapeutic strategies, such as PDT, sonodynamic therapy (SDT), chemotherapy, RT, starvation therapy, and immunotherapy. Besides, nanozyme-based catalysis monitoring and tumor diagnosis were reported.

### Catalysis Monitoring and Tumor Diagnosis In Vivo

**Fig. 12:**
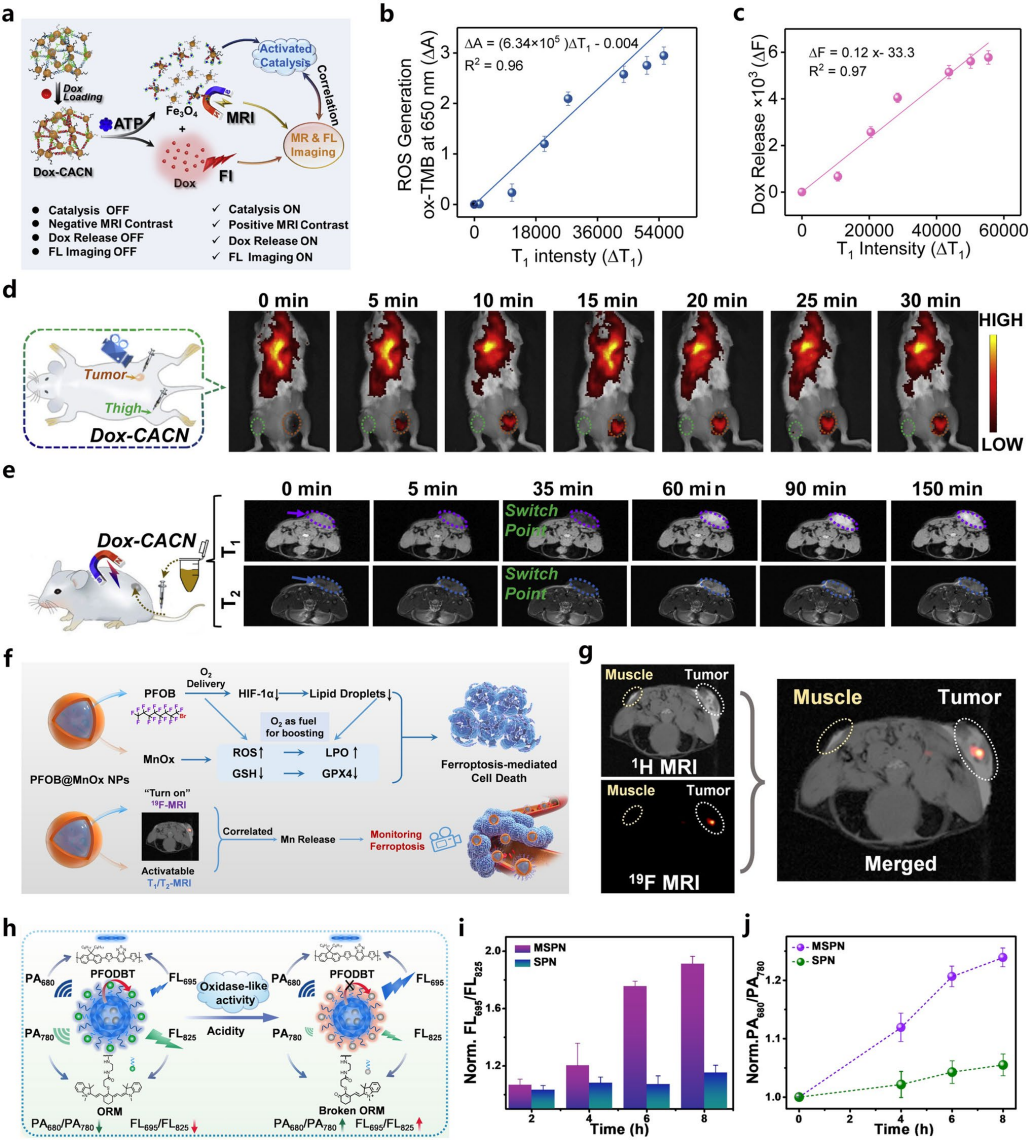
**a** Schematic diagram of Dox-CACN for switchable MRI precise monitoring of ferroptosis-related collaborative treatment. **b, c** Correlations between ROS generation and released Dox in Dox-CACN and MRI signals. **d**, **e** In vivo fluorescence and MRI of Dox-CACN [204]. Reproduced with permission. Copyright 2022, Elsevier Inc. **f** Schematic illustration of ^19^F/^1^H-MRI traceable perfluorocarbon@MnO_x_ for therapy. **g** T_1_-MRI and ^19^F-MRI of mice [206]. Reproduced with permission. Copyright 2022, Wiley–VCH GmbH. **h** Graphical illustration of MSPN with ratiometric NIRF-PA imaging for OXD-like activity monitor.** i**, **j** The normalized fluorescence intensity and PA ratios in vivo [210]. Reproduced with permission. Copyright 2021, Wiley–VCH GmbH

Exploring biological and pathological changes is significant for the early diagnosis and treatment of tumors. Nanozymes inherit the unique chemical and physical properties of nanomaterials, thus, are widely used for in vivo tumor imaging through various imaging techniques, mainly including fluorescence [196], PA [197, 198], ultrasound [199], photothermal [200], computed tomography (CT) [201], and magnetic resonance imaging (MRI) [202, 203]. To ensure the accuracy of diagnosis and the efficiency of cancer treatment, a visible treatment approach is expected by using in vivo imaging technology to monitor cancer diagnosis and treatment in real-time or in situ, adjusting treatment plans in a timely manner based on feedback information from imaging equipment, controlling drug dosage and avoiding excessive or insufficient treatment. Song et al. have committed to this field for many years and have made significant progress [204–210]. They developed a DNA functionalized, Fe_3_O_4_ self-assembled, and co-activated catalytic nanoplatform (CACN), which produced ROS under the condition of adenosine triphosphate (ATP) and slight acid, triggered specific ferroptosis/chemotherapy, and achieved accurate monitoring of treatment process through the switchable MRI (Fig. 12a) [204]. After loading with doxorubicin (Dox), the formed Dox-CACN underwent disassembly under ATP triggering, released DNA functionalized Fe_3_O_4_ nanoparticles and Dox, and triggered a transformation of MRI signal from T_1_-T_2_ double negative (darkened) to double positive (brightened). Under the triggering of a series of ATP concentrations, the disassembly of Dox-CACN triggered T_1_ and T_2_ signal enhancement (brightened). Under slightly acidic conditions, the production of ROS increases with the increase of ATP concentration. The precise release of fluorescence signals of Dox also gradually increases with ATP. By comparing the MRI signal, ROS production, and Dox fluorescence, we can find that there is a linear correlation between them (Fig. 12b-e). Therefore, we can monitor the treatment related to ferroptosis caused by ROS at the living level through the combination of MRI signal and Dox fluorescence.

Song et al. also revealed the MRI tracing of MnO_x_-coated perfluorocarbon (PFOB@MnO_x_) to overcome tolerance to hypoxia-induced ferroptosis therapy (Fig. 12f) [206]. Under the influence of TME, Mn ions are orderly dissociated from PFOB@MnO_x_, and the released Mn ions can change the ^1^H/^19^F-MRI signal, which can detect the release of Mn ions in the TME through changes in the signal in the tumor (Fig. 12g). The release of Mn ions is closely related to the occurrence of subsequent ferroptosis, thereby monitoring the treatment process in real-time. They also developed an intelligent nanozyme of MSPN, which contains mixed-valent MnO_x_, semiconductor polymer (PFODBT), and OXD-responsive molecule (ORM) through amide bonds (Fig. 12h) [210]. The specific catalytic therapy can be triggered by acidic TME due to the OXD-like activity of MnO_x_. Meanwhile, the catalytic activity of MSPN can be reported in real time through the output of ratiometric near-infrared fluorescence (NIRF) and PA signals. When ORM is destroyed by Mn^III^ under acidic conditions, the fluorescence intensity of MSPN decreases at 825 nm and increases at 695 nm due to the effective fluorescence resonance energy transfer between PFODBT and ORM, resulting in proportional NIRF signal output (FL_695_/FL_825_) (Fig. 12i). At the same time, the PA signal of PFODBT at 680 nm as internal reference almost remained unchanged, while the responsive PA signal of ORM at 780 nm decreased, resulting in the proportional output PA signal (PA_680_/PA_780_) (Fig. 12j). Therefore, MSPN possesses OXD-like activity for in vivo tumor treatment and real-time monitoring of the catalytic efficiency by integrating proportional signals from dual-mode NIRF-PA imaging.

**Fig. 13:**
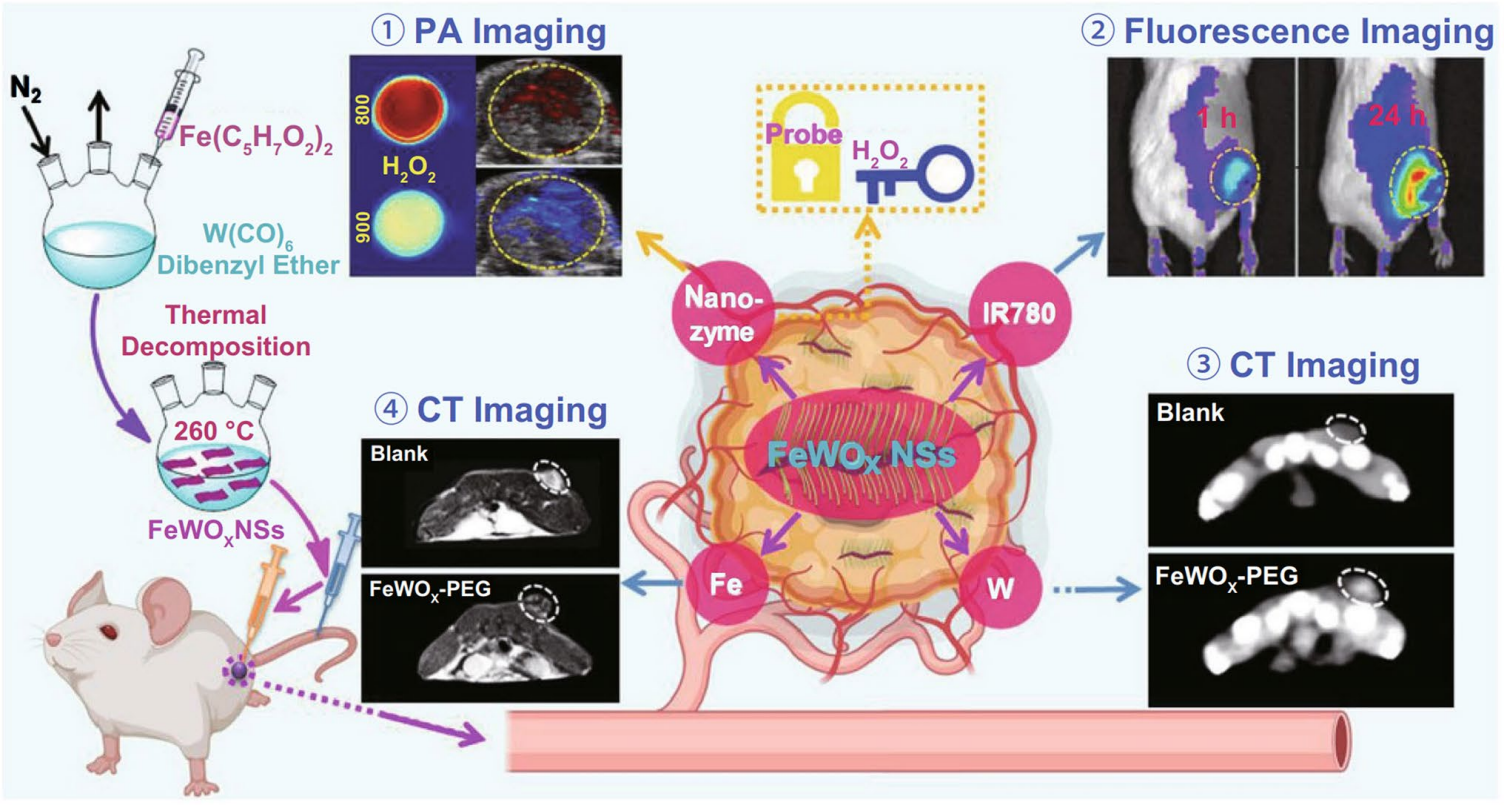
FeTIR nanoprobes with POD-like activity for multimodal tumor imaging [81]. Reproduced with permission. Copyright 2020, Wiley–VCH GmbH

Most imaging strategies rely on absolute intensity-dependent single signal acquisition, which may be influenced by experimental or physiological factors unrelated to the target, thus, resulting non-specific and misleading images to increase false positives. It is worth noting that the ratiometric method can overcome these problems. Ratiometric measurement provides built-in self-calibration for signal correction, thereby achieving more sensitive and reliable detection [211]. The NIRF imaging exhibits the advantages of high sensitivity and fast response [212, 213], and PA imaging has high imaging depth and spatial resolution in the body [214]. NIRF combined with PA imaging probes with proportional signals is beneficial to accurately monitor the catalytic activity of nanozymes in the body, achieving precise and personalized cancer treatment. Therefore, combining the unique enzyme simulation activity and an intelligent cancer diagnosis system based on nanozymes can be developed. For example, Cheng et al. prepared a FeTIR nanoprobe by loading both TMB and IR780 dye onto FeWO_x_ nanosheets for multimodal imaging [81]. Because of the strong X-ray attenuation effect of the W element, excellent fluorescence performance of IR780, and high MRI contrast of Fe element, the nanoprobes are suitable for CT, fluorescence, and MRI of tumors (Fig. 13). Since the H_2_O_2_ concentration is high in the TME, the loaded TMB will be oxidized in tumor, resulting in a significant increase of PA signal at 900 nm (PA900). By using the PA signal (PA800) of loaded IR780 at 800 nm as an internal reference, FeTIR nanoprobes can be used for ratiometric PA imaging based on the ratio of PA900 to PA800.

### Nanozyme for Synergistic and Cascade Catalytic Therapy

In organisms, many life activities can be achieved by various biochemical processes through a series of enzyme catalysis in cells in a cascade manner [215]. The catalytic performance of enzyme reactions in biological systems can be improved effectively due to the enzyme cascade reactions with high local concentrations of intermediates, reduced diffusion barriers, and high mass transfer efficiency [184]. However, due to the inherent limitations of natural enzymes, the enzyme cascade reaction system exhibits strict reaction conditions and poor stability, which greatly hinders further application in the biomedical field. As a substitute for enzymes, different nanozymes have different enzyme-like activities. When multiple nanozymes are integrated into one platform, a cascade catalytic system is constructed to effectively improve catalytic efficiency. In addition, some nanozymes can simulate various enzyme activities by themselves, providing new opportunities for constructing biomimetic self-cascade reaction systems. In cascade pathway, the product of one enzyme is usually served as the substrate of another enzyme. For example, GOx or GOx-like nanozymes can initiate reactions by catalyzing the production of significant H_2_O_2_ molecules from glucose within tumors. Subsequent catalytic reactions involving nanozymes with H_2_O_2_ molecules as substrates to generate ·OH through POD-like activity. Thus, highly toxic ·OH can be used for anti-tumor therapy or biosensing [216]. In addition, the inherent enzymatic catalysis properties of nanozymes, such as POD-like, CAT-like, OXD-like, and SOD-like activities, have been widely explored, which can effectively trigger reaction sequences and provide great possibilities for cascade reactions.

**Fig. 14:**
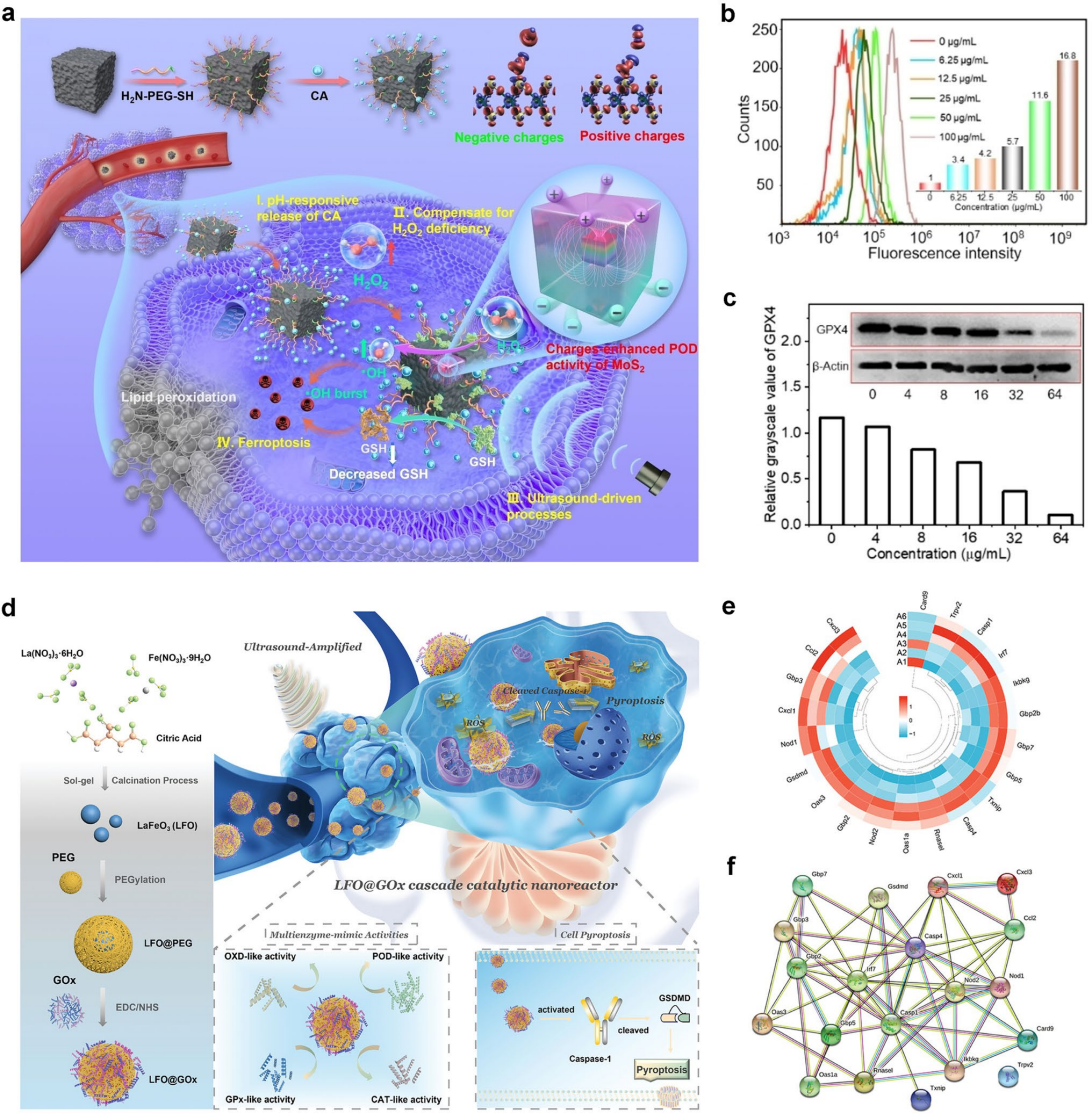
**a** Schematic diagram of synthesis and piezoelectric catalytic mechanism of BTO/MoS_2_@CA. **b, c** Flow cytometric analysis of ROS and western blot analysis of GPX4 with different BTO/MoS_2_@CA concentrations [217]. Reproduced with permission. Copyright 2022, Wiley–VCH GmbH. **d** LFO@GOx synthesis process and induction of pyroptosis as a cascaded catalytic nanoreactor for ultrasound-enhanced enzymatic therapy. **e** Ring numeric heatmap. **f** Protein–protein interaction network [218]. Reproduced with permission. Copyright 2023, Wiley–VCH GmbH

In recent years, on the basis of this background, more and more cascade theranostic nanosystems have been designed to optimize the treatment effectiveness. Chen et al. developed a cascade nanocatalyst (BTO/MoS_2_@CA) by combining piezoelectric barium titanate (BTO), MoS_2_ nanosheets with few layers, and PEG-modified cinnamaldehyde (CA) (Fig. 14a) [217]. The modification is pH responsive, thus, the therapy can be initiated by acidic TME and ultrasound. At the beginning, the CA is released to catalyze the production of a large amount of H_2_O_2_ in acidic TME, in order to improve tumor selectivity and reduce side effects. After that, the generated H_2_O_2_ undergoes a catalytic reaction by down-stream POD-like BTO/MoS_2_, releasing highly toxic ·OH radicals under ultrasound (Fig. 14b). Need to mention, the ability to consume GSH through sulfhydryl binding leads to a decrease in GPX4 expression due to the amplified oxidative stress (Fig. 14c), which further induces imbalance in redox homeostasis and ferroptosis in tumor cells. By integrating H_2_O_2_ self-supply and enhanced enzyme activity, BTO/MoS_2_@CA displays excellent specificity and synergy in tumor treatment.

Chen and co-workers reported an ultrasound-enhanced cascade catalytic therapy based on LaFeO_3_ (LFO) perovskite nanocrystals to improve the generation rate of harmful ROS and induce pyroptosis (Fig. 14d) [218]. The LFO nanocrystals exhibit OXD-like and POD-like activities to promote ROS generation, CAT-like activities involved in reversing hypoxia, and GPX-like activities related to GSH consumption. Need to mention, the above multi-enzyme catalytic reactions require the assistance of high levels H_2_O_2_, while the H_2_O_2_ content in TME is not sufficient to achieve desired therapeutic effects. Accordingly, GOx is introduced as a reactant for enzyme kinetic reactions in the cascade catalytic reaction process, serving as a tumor-specific H_2_O_2_ generator. Exogenous ultrasound induces significant tumor inhibition by increasing the conversion rate of intermediate complexes to Fe(II). Encouragement by the in vitro and in vivo proliferation-inhibitory abilities, a comprehensive and sequential validation of the increased ROS and induced pyroptosis process of the ROS-TXNIP-NLRP3-GSDMD pathway was conducted. As shown in Fig. 14e, f, the expression of major genes involved in pyroptosis was highlighted by the ring numeric heatmap and protein–protein interaction network. Song et al. designed a synergistic and self-cyclic catalysis system by combing GOx and Cu_3+x_(PO_4_)_2_ nanozyme through biomineralization [219], which experienced consumption of glucose, depletion of GSH, and generation of ·OH/^1^O_2_.

Fan and co-workers proposed a POD-like pyrite nanozyme with ultra-high H_2_O_2_ affinity, and the catalytic activities were 4144 and 3086 times higher than that of classical Fe_3_O_4_ nanozymes and natural HRP, respectively [220]. The pyrite nanozyme also has activity similar to GSH oxidase, which can catalyze the oxidation of GSH and produce H_2_O_2_. Therefore, the pyrite nanozyme with dual activity constituted a self-cascading platform that can generate abundant ·OH and consume GSH, thereby inducing tumor cell apoptosis and ferroptosis. At the same time, the pyrite nanozyme has good tumor-specific toxicity and biodegradability, maintaining good biosafety during the treatment process. The results indicate that high-performance pyrite nanozymes are effective therapeutic reagents and contribute to the further development of nanozyme-based catalytic therapy.

Nanozyme-driven chemodynamic therapy (CDT) refers to a highly tumor-specific cancer treatment mode mediated by a nanozyme with POD-like activity, which can in situ catalyze the conversion of endogenous H_2_O_2_ into highly toxic ∙OH to induce apoptosis and necrosis of cells. Although the inherent POD-like activity of many nanozymes has been revealed and used for cancer diagnosis and treatment, including ferromagnetic nanoparticles (γ-Fe_2_O_3_ or Fe_3_O_4_) [221, 222], vanadium oxide [223, 224], copper oxide [225, 226] and CeO_2_ [113, 227], their inherent low catalytic repetition rate and insufficient catalytic performance make them difficult to achieve ideal therapeutic effects during the treatment process. Besides, due to the limitations of endogenous H_2_O_2_ and low catalytic efficiency of the catalyst, CDT is unable to achieve satisfactory anti-tumor efficacy. We propose a multifunctional γ-Fe_2_O_3_-GOx-DMSN nanocatalyst for TME specific response catalysis and magnetic targeting of tumors, which integrate CDT and PTT together to achieve enhanced cascade catalytic therapy effects [221]. After tail vein injection, the *γ*-Fe_2_O_3_-GOx-DMSN can target and enrich at the tumor site under magnetic targeting. The loaded GOx can specifically catalyze the consumption of glucose, causing an increase in H_2_O_2_ concentration and a decrease in pH value. The generated H_2_O_2_ can be disproportionated to produce a large amount of ·OH by the POD-like activity of γ-Fe_2_O_3_ to induce oxidative damage to tumor cells. More importantly, the strong light absorption ability in the near-infrared (NIR) region exhibits outstanding photothermal efficiency under 808 nm laser irradiation, which can enhance tumor catalytic therapy through PTT synergy. This strategy of constructing cascade catalytic reactions in situ within tumor cells provides an important paradigm for achieving efficient tumor catalytic therapy.

### Nanozyme-Assisted PDT/SDT

**Fig. 15:**
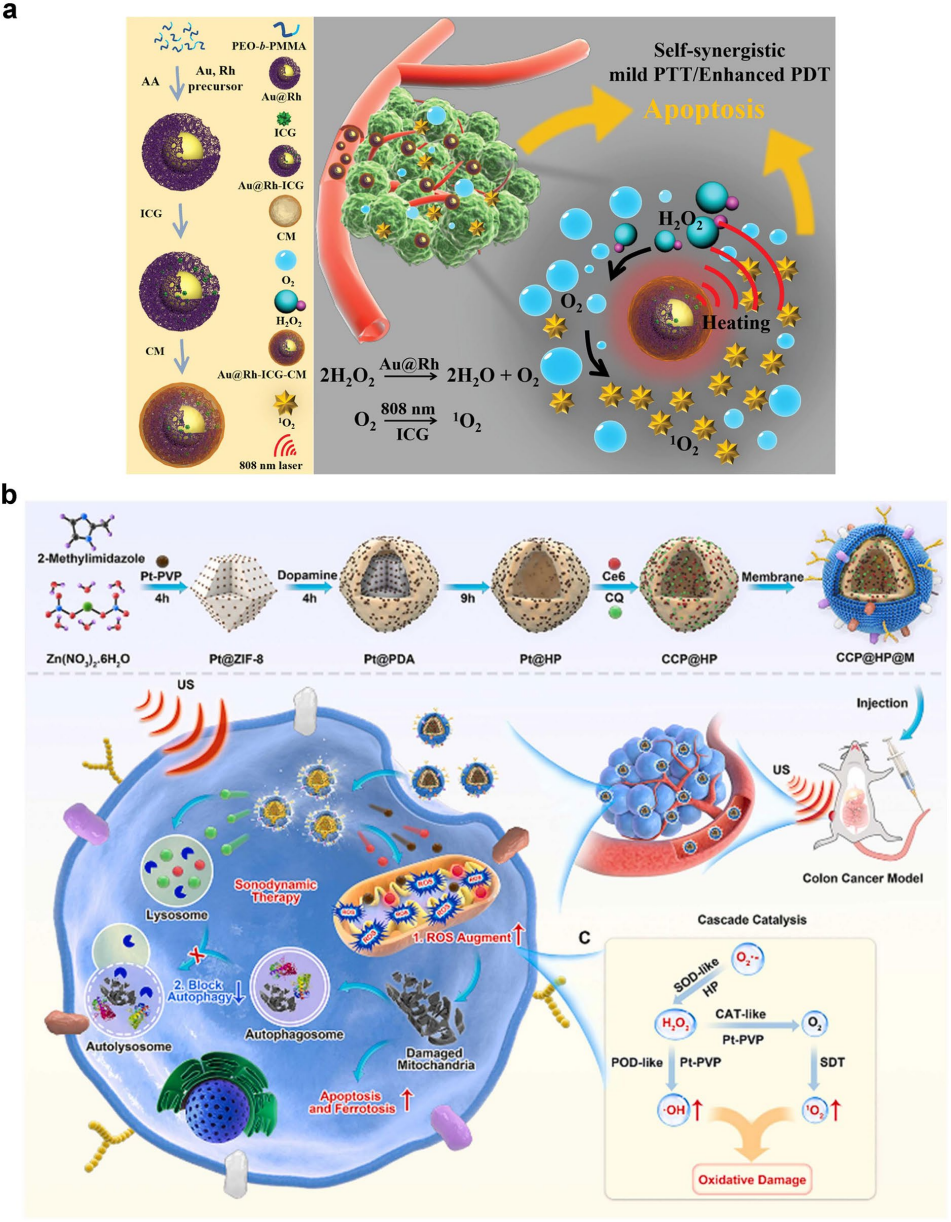
**a** The schematic diagram of the synthesis of Au@Rh-ICG-CM and the main therapeutic mechanism for cancer [86]. Reproduced with permission. Copyright 2020, WILEY–VCH Verlag GmbH & Co. KGaA, Weinheim. **b** Schematic diagram of the synthesis of CCP@HP@M and autophagy blockade achieved in SDT for the treatment of colorectal cancer [87]. Reproduced with permission. Copyright 2023, Elsevier Ltd

PDT is a therapeutic approach based on the interaction of light, photosensitizers, and ROS. When a photosensitizer absorbs light energy at a specific wavelength, the energy level transition occurs to generate electrons which may combine with surrounding O_2_ and other small molecules to generate ROS species of ·OH, ^1^O_2_, O_2_^·−^, etc. The ROS can oxidize biological macromolecules including lipids, proteins, and DNA in cells, thereby inducing tumor cell death [228]. As a time and space-controllable, non-invasive, and efficient phototherapy method, PDT has become one of the favored treatment strategies in medical research. In solid tumors, due to the vigorous metabolism of tumor tissue, actually, most tumor cells are in a hypoxic environment, which indirectly limits the production of ROS during the PDT process and hinders the PDT effect. Due to abnormal physiological metabolic processes in tumors, such as the overexpression of NADPH oxidase, production of H_2_O_2_ with a rate of 5 nmol per hour per 10^5^ cells, and increase in H_2_O_2_ content in the TME [229], the development of nanomaterials that can catalyze the conversion of endogenous H_2_O_2_ to O_2_ is receiving increasing attention. For instance, Wang et al. designed Au@Rh core–shell nanostructure loading with photosensitizer indocyanine green (ICG), then the tumor cell membrane was coated to construct the Au@Rh-ICG-CM nanocomposite (Fig. 15a) [86]. The Au@Rh-ICG-CM nanosystem can rapidly decompose endogenous H_2_O_2_ to generate O_2_ in neutral or acidic environments. The loading efficiency of ICG can be improved and the activity during physiological transportation can be stabilized due to the porous structure combined with the capture ability of tumor cell membranes. Moreover, under 808 nm irradiation, the mild photothermal effect is beneficial for the catalytic decomposition of H_2_O_2_ and promotes cellular uptake of ICG. Because of the homologous binding ability of tumor cell membrane, the nanocomposite can selectively accumulate to the tumor site, thus improving the specificity of PDT. In vitro and in vivo anti-tumor studies show that Au@Rh-ICG-CM is a multifunctional oxygenator in tumors and significantly enhances PDT efficacy under hypoxic conditions.

SDT is an emerging non-invasive tumor treatment method, which mainly uses low-intensity ultrasound to stimulate the sonosensitizer enriched in the tumor site, thus, producing ROS, mechanical damage or thermal damage to induce irreversible damage to tumor cells. The SDT triggered by ultrasound possesses the superiorities of high tissue penetration up to several tens of centimeters and high spatiotemporal selectivity, making it an emerging method for treating deep solid tumors [230, 231]. However, the therapeutic effect of O_2_-dependent SDT is often hindered by the low ROS generation efficiency and the activated protective autophagy. Zhang et al. prepared a biomimetic cascade CCP@HP@M by loading autophagy inhibitor chloroquine and Ce6 sonosensitizer into Pt-doped polydopamine nanocarrier, and then modified with homologous tumor cell membranes (Fig. 15b) [87]. Among them, polydopamine exhibits SOD-like activity, which converts O_2_^·^^−^ into O_2_ and H_2_O_2_. Pt nanozymes further catalyze the conversion of excess H_2_O_2_ to ·OH and O_2_. As a pH-responsive nanocarrier, the loaded Ce6 and chloroquine can be released from CCP@HP@M in acidic conditions. Under ultrasound irradiation, the released Ce6 produces toxic ROS, and the released chloroquine inhibits the protective autophagy pathway, reducing the resistance of colon cancer to SDT. Combining ROS generation and autophagy inhibition, this cascaded nanoreactor may induce cell apoptosis and ferroptosis, ensuring the efficiency of SDT and expecting to be a new strategy for tumor treatment.

### Nanozyme-Assisted Chemotherapy

**Fig. 16:**
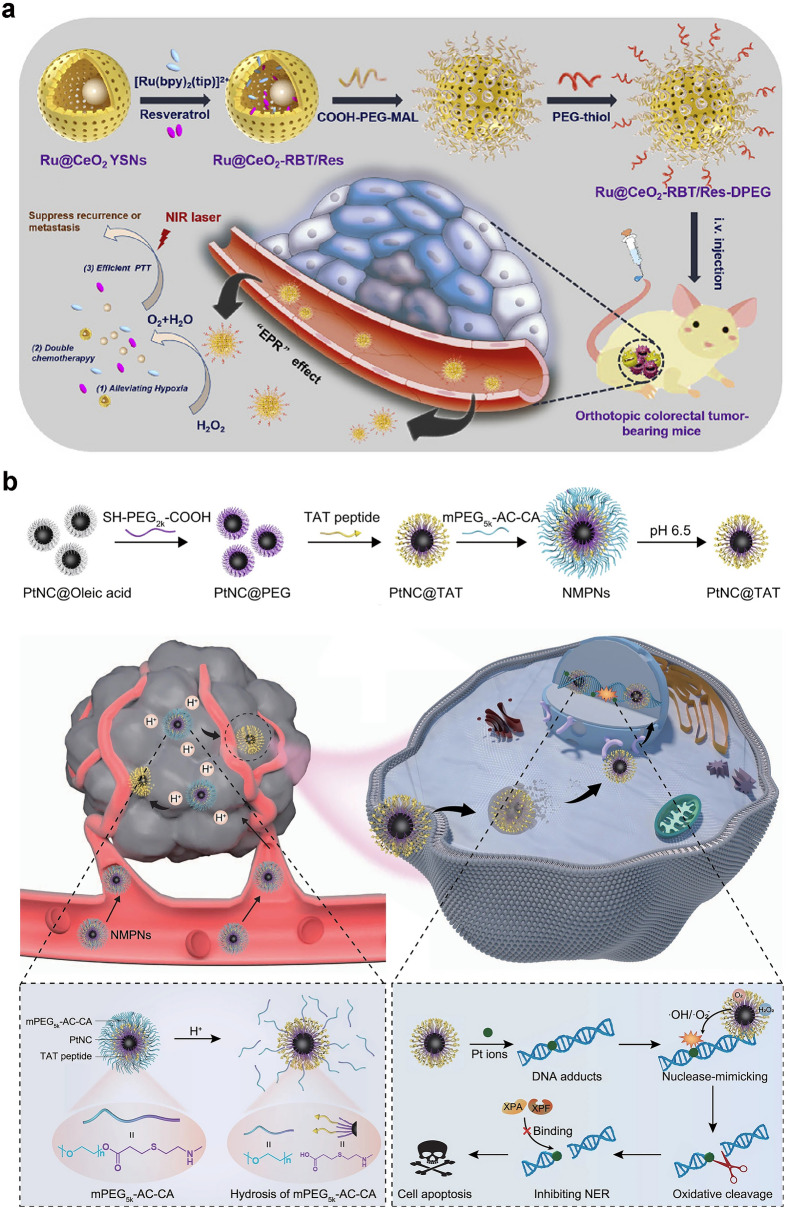
**a** Schematic diagram of the preparation process and therapeutic mechanism of Ru@CeO_2_-RBT/Res-DPEG dual-drug nanozyme [234]. Reproduced with permission. Copyright 2020, Elsevier Ltd. **b** Schematic diagram of the synthesis of NMPNs and NMPNs-mediated DNA platinization and oxidative cleavage to overcome Pt resistance of cancer [235]. Reproduced with permission. Copyright 2022, Springer Nature

Chemotherapy is the treatment method by using chemical drugs, in order to prevent the proliferation, infiltration, metastasis, and ultimately kill cancer cells. Chemotherapy remains one of the mainstream methods for cancer treatment. Numerous studies have shown that solid tumor tissue has physiological characteristics such as hypoxia and weak acidity compared to normal tissue. In particular, there is an inseparable relationship between the hypoxic environment of tumor tissue and tumor multidrug resistance [232]. The generation of hypoxia in tumor tissue is generally believed to be insufficient oxygen diffusion and supply in areas far from capillaries in solid tumor tissue, as well as the rapid proliferation of tumor cells that consume massive oxygen. Research has shown that PTT, as a novel local non-invasive treatment method, can significantly enhance the effectiveness of cancer treatment in combination with chemotherapy [233]. In addition, overcoming tumor hypoxia by catalyzing endogenous H_2_O_2_ generation through nanozymes is considered as an effective strategy to enhance the anti-tumor effects of various chemical drugs. Accordingly, Liu et al. designed and synthesized yolk-shell Ru@CeO_2_ nanozymes with excellent photothermal conversion performance and CAT-like activity [234]. By loading two anti-tumor drugs of ruthenium complex (RBT) and resveratrol (Res) onto Ru@CeO_2_, followed by constructing of PEG layer, a dual drug-delivery Ru@CeO_2_-RBT/Res-DPEG system with on-demand release was obtained (Fig. 16a). The Ru@CeO_2_ nanozymes with CAT-like activity can catalyze endogenous H_2_O_2_ to produce oxygen and alleviate tumor hypoxia. Compared with RBT or Res alone, they can significantly induce tumor cell apoptosis. Combined with the photothermal characteristics, yolk-shell Ru@CeO_2_ nanozyme achieved effective synergy between dual-chemotherapy and PTT, showing significant anti-tumor effects in vivo and inhibiting the metastasis and recurrence of subcutaneous colorectal cancer effectively.

Resistance of cancer cells occurs during clinical chemotherapy based on platinum drugs almost inevitably. Lots of Pt-based nanomedicines, such as Pt-loaded nanocarriers and metal Pt, have been designed for targeted delivery and combination therapy to overcome tumor resistance [236, 237]. Most Pt-based compounds and nano-drugs play an anticancer role by inducing Pt–DNA adduct [238, 239]. The treatment will activate the DNA-damage response system in tumors to remove Pt–DNA adduct [240]. Then, the DNA repair pathways, mainly including nucleotide excision repair (NER) and base-excision repair play a part in repairing DNA damage [241]. It is worth noting that the NER pathway is a typical pathway leading to Pt resistance, significantly impairing the therapeutic effect of Pt drugs [242]. Recently, Ling et al. designed a nuclease-mimic Pt nanozymes (NMPNs) (Fig. 16b) [235]. The nanozyme can target tumor nuclei to trigger Pt–DNA adduct effectively, meanwhile, change the DNA conformation required by NER to conquer Pt resistance. NMPNs targeted to tumor nuclei also release Pt ions to conquer the generation of Pt–DNA adduct. On the other hand, NMPNs play an efficient OXD-like and POD-like activity to produce ROS (O_2_^·−^ and ·OH), which further cooperate with the Pt ions to start the oxidative cleavage of double-stranded DNA, finally, blocking the DNA bending demanded by NER. Overall, the nuclease-like NMPNs on behalf of the new generation of Pt-based drugs may damage DNA and alter the DNA conformation, opening up new directions for clinical DNA-damaged chemotherapy.

As discussed above, single-atom nanozymes with POD-like activity can alter cellular redox balance and exhibit promising application prospects in tumor therapy. However, the insufficient H_2_O_2_ content inside solid tumors greatly limits the efficacy of single-atom nanozymes. To solve the problems, Fan and co-workers encapsulated single-atom nanozymes and camptothecin chemotherapeutic drugs in injectable hydrogels to form a light-controlled oxidative stress amplification system [243]. This system can enhance the therapeutic effect of single-atom catalysis. It has been found that immunosuppressed “cold” tumors can be transformed into “hot” tumors with excellent synergistic immunotherapy ability. It has broad application prospects and high clinical translatability in the field of digestive tract tumor treatment.

### Nanozyme-Assisted RT

**Fig. 17:**
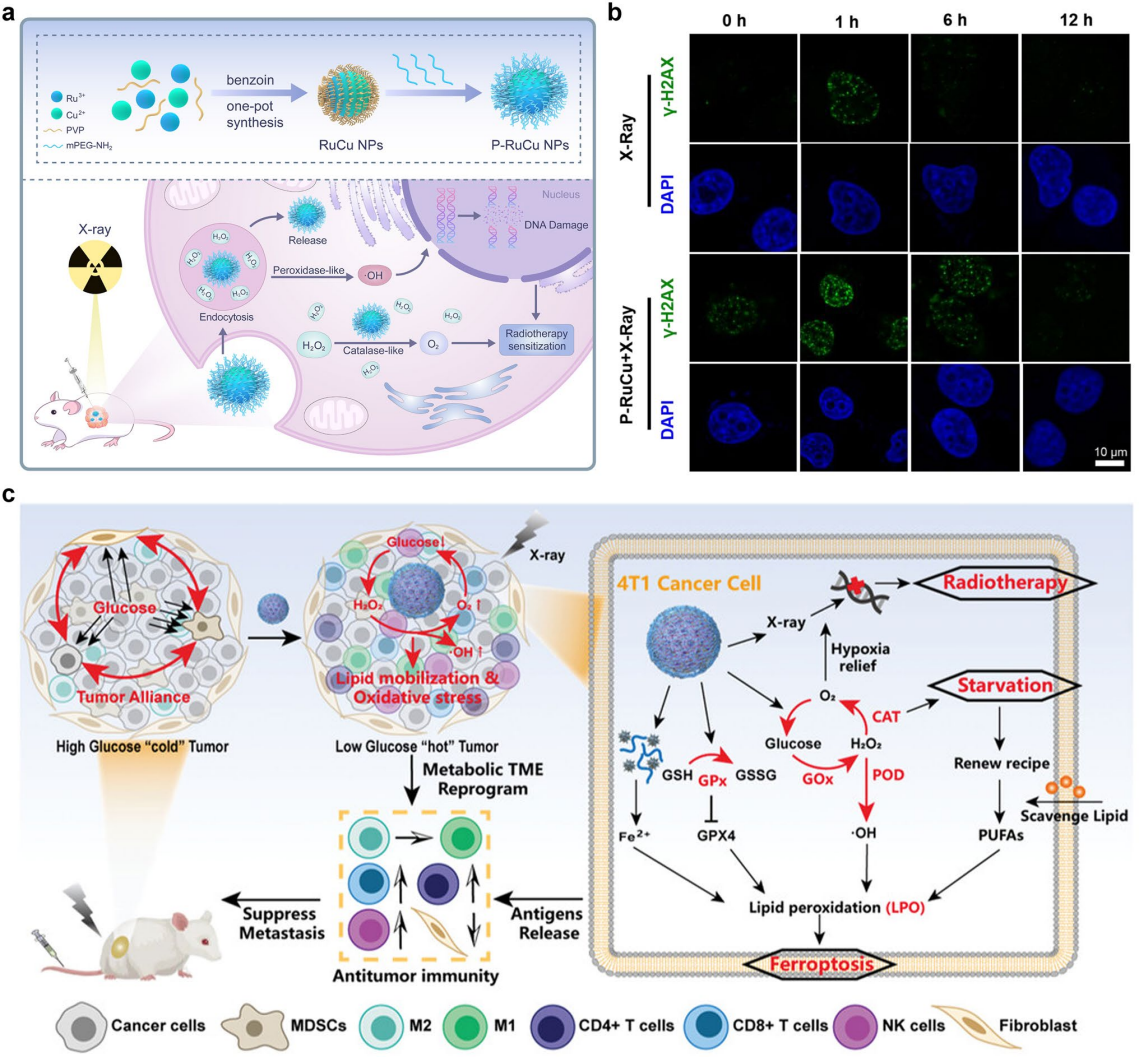
**a** Schematic diagram for synthesis and enhanced cancer RT of P-RuCu nanozyme. **b** Confocal fluorescence images of MDA-MB-231 cells stained with γ-H2AX (green) and DAPI (blue) [88]. Reproduced with permission. Copyright 2022, Elsevier Ltd. **c** Schematic diagram of tumor metabolism reprogramming for enhanced radioimmunotherapy of PdPtAu@TF [89]. Reproduced with permission. Copyright 2023, Wiley–VCH GmbH. (Color figure online)

Tumor RT is a local treatment strategy assisted by various radiations of *α*, *β*, and* γ*-rays emitted from radioactive isotopes, X-rays, proton beams, electron beams, and so on, to kill tumors. RT is a main method for tumor treatment in medical practice, and has a glorious future, while the efficacy is usually limited by the limitations of radiation energy, which can easily cause side damage to normal tissues [244, 245]. In addition, tumor hypoxia microenvironment, up-regulation of tumor cell antioxidant system, and DNA repair protein are the main reasons for RT resistance [246, 247]. Therefore, development of efficient and low-toxicity radiosensitizer is of great significance in overcoming radiation resistance. The interaction of nanozyme and TME to achieve tumor synergistic therapy is an effective strategy to improve the efficacy and safety of RT. Fan and co-workers synthesized ferrihydrite with CAT-like activity to produce O_2_ and enhance RT, and the 2-line ferrihydrite exhibited the highest CAT-like activity [248]. The ferrihydrite is instructive, as it showed no POD-like, OXD-like and SOD-like activities in TME. Zhang et al. constructed a bimetallic nanoparticles (RuCu NPs) as a novel radiosensitizer (Fig. 17a) [88]. The element Ru with a high atomic number absorbs X-rays and produces ROS to enhance radiosensitivity. In addition, RuCu NPs exhibit both CAT-like and POD-like activities. On the one hand, RuCu NPs interact with the TME to produce ·OH and enhance the tumor therapeutic effect. On the other hand, RuCu NPs play CAT-like activity to catalyze H_2_O_2_, produce O_2_, and alleviate tumor hypoxia, demonstrating a significant therapeutic effect in the MDA-MB-231 breast cancer model. As shown in Fig. 17b, compared to the group treated with X-rays alone, PEGylated RuCu (P–RuCu) was observed to have the largest *γ*-H2AX foci density, indicating an enhanced therapeutic effect of P–RuCu nanozyme on RT.

Since cancer cells undergo metabolic reprogramming in the process of tumor occurrence and development to meet their increased requirement for bioenergy and biosynthesis, meanwhile, reduce the oxidative stress demand for cancer cell survival and proliferation, a TME with high antioxidant and glucose addiction has formed, which greatly hindered cancer treatment [249, 250]. In view of the unique metabolic characteristics of TME, various emerging treatment strategies, such as starvation therapy, PDT, and CDT, have arisen extensive research and made significant progress [251, 252]. However, due to the metabolic flexibility and plasticity, tumor metabolism will be changed by fluctuations of ROS or nutrients, inducing therapeutic resistance [253]. Therefore, regulating the metabolic activity and plasticity of tumors may be an effective strategy to improve the traditional RT efficacy. Zhang et al. synthesized mesoporous Pd/Pt/Au nanoparticles by using surfactant-directing method, and coated them with tannic acid-iron ion (Fe^III^-TA) network to form pH-responsive PdPtAu@TF [89]. The PdPtAu nanoparticles have GOx-, CAT-, POD-, and GPX-like activities (Fig. 17c). The Fe^III^-TA coating can be degraded under an acidic environment, while the enzymatic catalytic activity would not be destroyed. The prepared PdPtAu@TF competes with cancer cells to react with glucose and generate H_2_O_2_, making the cells fall into a “ hungry state”, thereby enhancing the uptake and utilization of “reserve energy” lipids. At the same time, PdPtAu@TF catalyzes the decomposition of H_2_O_2_ within cells to generate ROS and O_2_, consuming the antioxidant GSH, thereby, inducing ferroptosis and sensitizing RT in cancer cells. There were several ways to initiate the ferroptosis mechanism. On the one hand, based on the CAT, POD, and GOx combined catalytic therapy, PdPtAu@TF could produce a large amount of ROS to accumulate lipid peroxidation and induce ferroptosis. On the other hand, as an essential cofactor, GSH was depleted to inhibit the expression of GPX4, and prevent the reduction of the toxic lipid peroxidation to induce ferroptosis. Besides, the starvation may enhance the ferroptosis by “Renew recipe” process. Meanwhile, RT effectively activates the immune response of mice and promotes macrophage polarization towards the M1 phenotype. The PdPtAu@TF combined RT triggers a stronger anti-tumor immune response than single therapy, which systematically regulates adaptive and innate immunity, transforming immune “cold” tumors into “hot” tumors, thereby inhibiting tumor metastasis.

### Nanozyme-Assisted Starvation Therapy

“It is hard to labor with an empty belly”. People are like this, and so are cells. Especially tumor cells, because of the abnormal growth and excessive proliferation, require more energy supply compared to normal cells. Tumor starvation therapy can be realized by cutting off the energy supply, which interferes with tumor angiogenesis, consumes glucose by GOx, or inhibits the function of glucose transporters on the surface of tumor cell membranes, in order to hinder the growth and reproduction of tumor cells and induce their death. It has become an efficient and safe treatment method in the field of anti-tumor research [254–256]. It should be emphasized that this energy cutoff is not a generalized approach that needs patients to reduce their intake of food or consume less nutritious foods, as it can result in insufficient energy for normal tissues and a decrease in immune function. Starvation therapy can inhibit the metabolic process of tumor cells selectively and specifically, achieving precise therapy against tumors. In recent years, encouraged by the thorough study of tumor growth mechanisms, starvation therapy combined with other methods such as catalytic therapy [257], PTT [258], PDT [259], and CDT [260] can synergistically inhibit tumor growth and achieve more efficient anticancer effect.

**Fig. 18:**
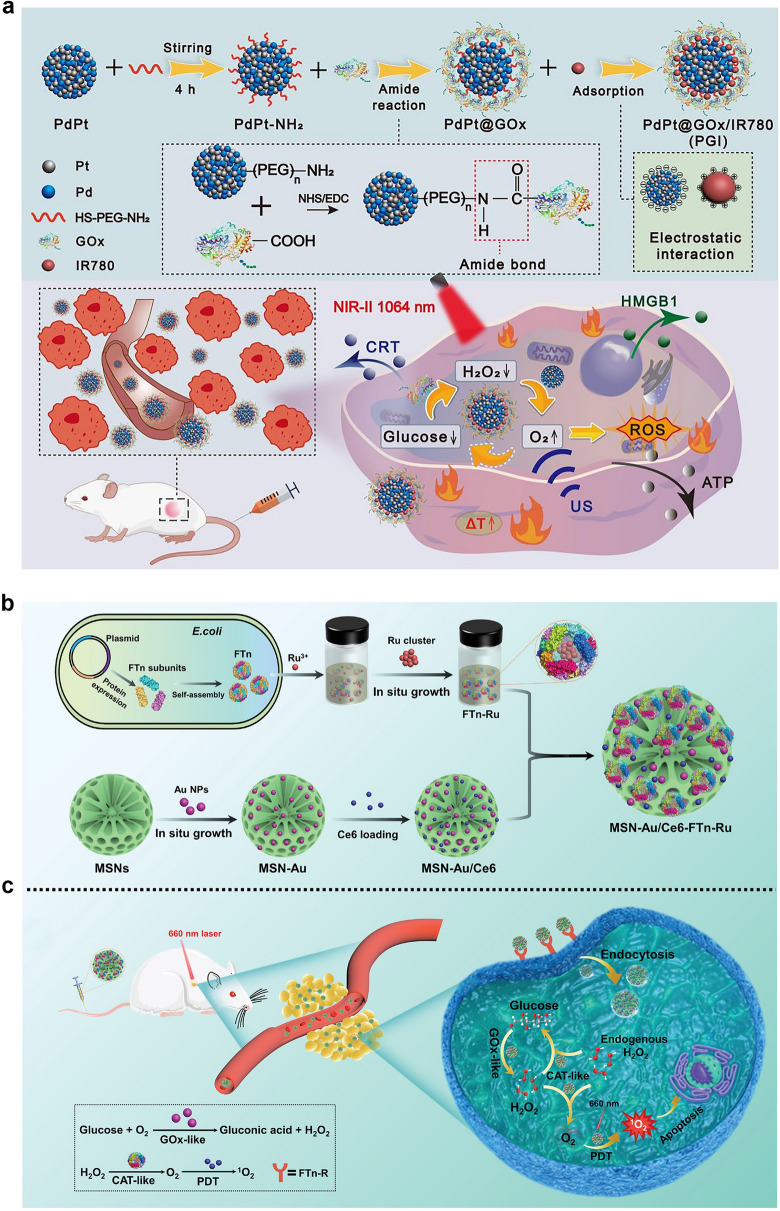
**a** Synthesis process of PGI and its application in photothermal enhanced tumor starvation therapy and SDT [257]. Reproduced with permission. Copyright 2022, Elsevier B.V. **b**, **c** Synthesis process of MSN-Au/Ce6-FTn-Ru and enhanced tumor targeting starvation and PDT mechanism [263]. Reproduced with permission. Copyright 2022, Wiley–VCH GmbH

The depletion of intracellular glucose to achieve tumor starvation has been proven to be a promising anticancer treatment strategy. However, the treatment effectiveness can be influenced significantly by the insufficient oxygenation in hypoxic tumor tissue. Xue et al. synthesized a bimetallic PdPt nanocatalyst. Then, GOx was fixed onto the surface of PdPt through covalent bonding, and the sonosensitizer IR780 was encapsulated through strong electrostatic interaction to form PdPt@GOx/IR780 (PGI) (Fig. 18a) [257]. The PGI was used for NIR-photothermal enhanced tumor starvation and SDT, supplemented by oxygen self-supply strategies. As a type of CAT-like nanozyme, PGI can alleviate tumor hypoxia by catalyzing the conversion of H_2_O_2_ into O_2_. In addition, tumor starvation therapy can be achieved by GOx mediated glucose consumption within tumors, since which blocks the necessary energy and nutrient supply. The produced H_2_O_2_ supplies local O_2_ continuously, which in turn further exacerbates glucose consumption. At the same time, with the help of sonosensitizer IR780, the relief of hypoxia promotes the production of ^1^O_2_, resulting in more effective SDT. On the other hand, the PTT under the second near-infrared (NIR-II) irradiation further induces tumor cell ablation, combining with tumor starvation and SDT to achieve complete tumor eradication. In addition, combined therapy causes strong immunogenic cell death, which is beneficial for overcoming immune suppression and enhancing anti-tumor immunity. The good tumor inhibitory effect of PGI has been confirmed in a tumor-bearing mouse model. Besides, natural GOx has limitations such as high cost, sensitivity to chemical modifications, and instability under harsh conditions. Utilizing GOx-like nanozymes to promote glucose catalytic oxidation and generate H_2_O_2_ for starvation treatment is an alternative strategy [216, 261]. Currently, research on phototherapy mostly focuses on the first near-infrared region (NIR-I, wavelength of 700–900 nm) with a tissue penetration depth of approximately 1 mm [262]. In recent years, research has shown that compared to the visible light region and the NIR-I region, the light in the NIR-II region with wavelength of 1000–1700 nm is less absorbed, scattered and reflected when it propagates in biological tissues such as skin, fat, and bone. At the same time, the background autofluorescence of biological tissues in this region is reduced, and the signal-to-noise ratio is improved [262]. It is particularly important that the NIR-II region allows for a higher maximum permissible exposure laser. Accordingly, NIR-II light can provide a deeper tissue penetration depth of approximately 5–20 mm, which is good for the practical application of phototherapy.

Huang et al. designed a protein corona cloaking-based nanoplatform (MSN–Au/Ce6–FTn–Ru) with cascaded catalytic reactions, by using human ferritin heavy chain nanocages (FTn) as protein corona and nanozyme as generator for synergistic tumor treatment [263]. As shown in Fig. 18b, after in situ growth of Au on mesoporous silica nanoparticles (MSN), Ce6 photosensitizers were connected through acylation reactions, and FTn-Ru was loaded through a simple mixing method. The design principles of this multifunctional nanoplatform are as follows: (1) Au nanoparticles in the surface/pores of MSN consume glucose to produce H_2_O_2_ for starvation treatment. (2) The FTn is genetically engineered, which can target hypoxic tumor cells actively and reduce protein corona formation derived by plasma effectively. (3) The Ru nanoclusters serve as a CAT-like nanozyme, promoting H_2_O_2_ to O_2_ and optimizing the O_2_-dependent PDT efficacy. By consuming endogenous glucose and H_2_O_2_ in the tumor, this nanozyme induces cascade and cyclic catalytic reactions, providing O_2_ for the production of cytotoxic ^1^O_2_ continuously (Fig. 18c). With the above advantages, the nanoplatform possesses effective tumor-killing ability in animal models.

Fan and co-workers developed a multifunctional FePGOGA nanozyme composed of Fe(III)-mediated oxidative-polymerization (FeP), GOx, and GAP19 peptides [264]. The FePGOGA nanozyme has excellent cascading POD and GSH oxidase mimicking activity, which can effectively catalyze the conversion of H_2_O_2_ to ·OH and convert GSH into oxidizing glutathione disulfide. In addition, loaded GOx made tumor cells starve and exacerbate tumor oxidative stress by decomposing glucose, while GAP19 peptide induced connexin 43 degradation to block hemichannels, thereby increasing intracellular ROS accumulation and reducing intracellular glucose transport. The ROS reacts with heat shock proteins, disrupting their structure and function, leading to tumor PTT at mild temperatures (≤ 45 °C). In vivo experimental results indicated that FePGOGA exhibited good curative effect on tumors under 808 nm light. In summary, this report proves that ablation of gap junction proteins successfully overcomes tumor resistance to ROS-assisted therapy and provides a novel regulator for inhibiting tumor self-protection during catalytic therapy, mild PTT, and tumor starvation.

### Nanozyme-Assisted Immunotherapy

Tumor immunotherapy is a treatment method, which restarts and maintains the tumor immune cycle, and restores normal anti-tumor immune response, thereby controlling and clearing tumors. Immunotherapy achieves effective clearance of tumors by activating its own anti-tumor immune system and inducing long-term immune monitoring effects in the body. It has become the most effective tumor treatment method after surgical treatment, RT, and chemotherapy [265]. Unfortunately, the response rate of tumor immunotherapy is low and needs to be improved. In most tumors, taking breast cancer as an example, immune checkpoint blockade (ICB) represented the response rate of tumor immunotherapy is less than 20% [266]. Therefore, immunotherapy combined with other treatments is commonly used in preclinical studies for tumor treatment, such as chemotherapy or RT [267]. However, chemotherapy or RT usually causes serious harm to the body, therefore, low-toxicity treatment methods combined with immunotherapy are crucial.

**Fig. 19:**
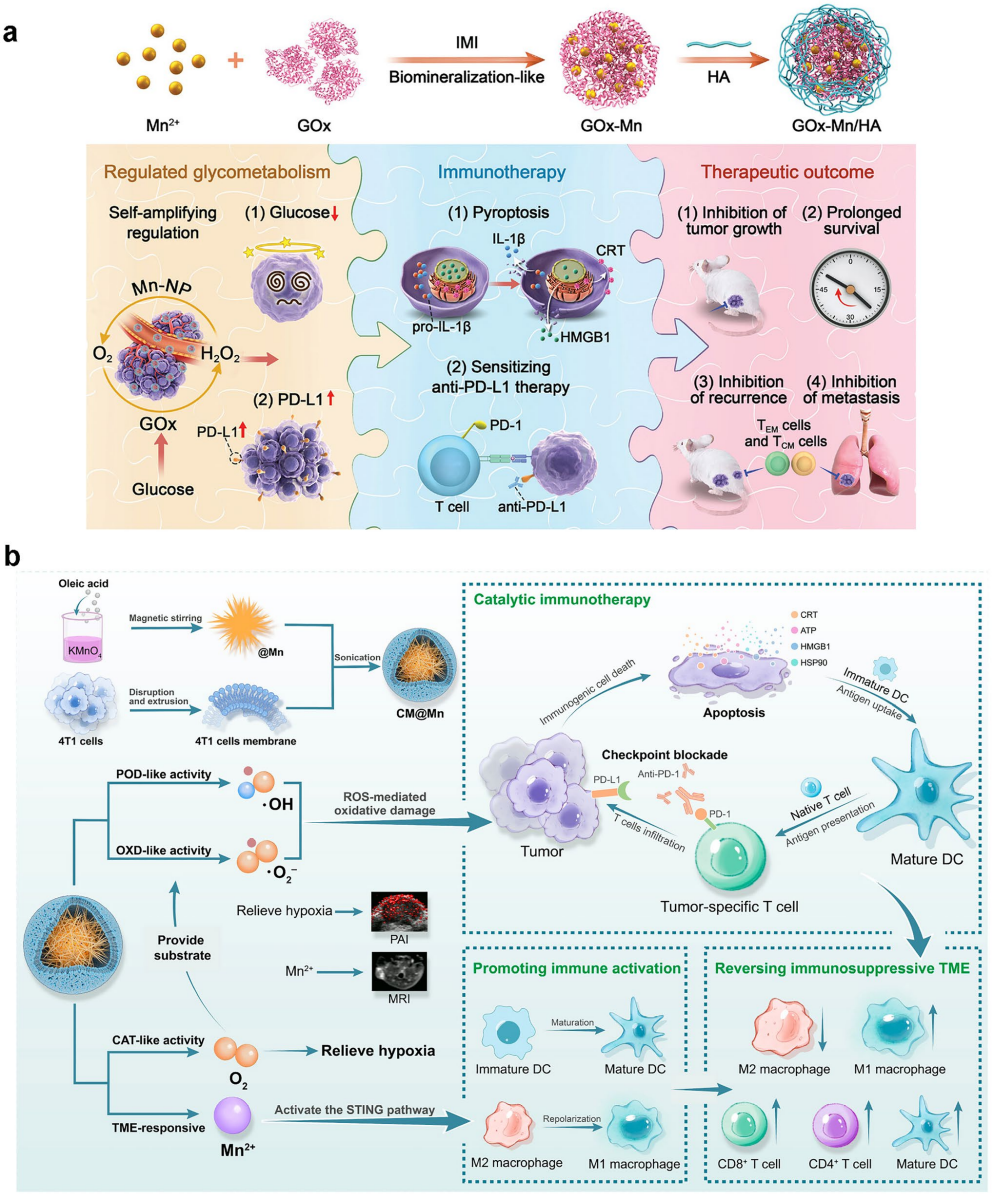
**a** Synthesis of GOx-Mn/HA and biomineralization of two-enzyme nanoparticles to regulate tumor glycometabolism and induce tumor pyroptosis and anti-tumor immunotherapy [272]. Reproduced with permission. Copyright 2022, Wiley–VCH GmbH.** b** Schematic diagram of CM@MnO_x_ preparation and treatment strategy of TME-activated and manganese-enhanced catalytic immunotherapy combined with PD-1 checkpoint blockade [273]. Reproduced with permission. Copyright 2022, American Chemical Society

Studies proved that tumor treatment by regulating metabolism is a promising method with good therapeutic effects and low side effects [268]. Glycolysis is the main form of tumor glucose metabolism. As a representative of ICB therapy, programmed death-ligand 1 (PD-L1) is associated with glycolytic activity [269]. Consuming glucose to enhance tumor glycolytic activity normally leads to more PD-L1 expression in tumor cells, hence, boosting anti-PD-L1 immunotherapy [270]. Besides, blocking PD-L1 may be a good choice to inhibit tumor glycolytic performance and activate T cells [271]. Accordingly, combining the ICB therapy with tumor glucose metabolism regulation can achieve synergistical therapeutic effects. Tian and co-workers adopted a biomineralization method to prepare GOx-Mn nanoparticles with double-enzyme activity (Fig. 19a) [272]. First, the manganese-containing nanozymes catalyze H_2_O_2_ to generate O_2_ at the tumor site. Then, the produced O_2_ promotes the glucose consumption of GOx and regulates glucose metabolism at tumor sites effectively. The generated H_2_O_2_ is also good for the catalytic reaction of GOx-Mn. Thus, the fusion of GOx and nanozymes enables the cascade catalytic reactions and cyclical magnification of glucose consumption. The glucose consumption leads to more PD-L1 expression in tumor cells, and then enhances the ICB therapy of PD-L1/PD-1. Besides, GOx-Mn was modified with hyaluronic acid (HA) to produce GOx-Mn/HA. Because of the specific binding between HA and CD44 on the surface of 4T1 cells, tumor-targeted delivery was achieved in breast cancer. This treatment strategy inhibits the development of tumors and increases the survival time of mice greatly. This combined treatment mode also exhibits an important immunological memory effect, which prevents tumor metastasis and recurrence successfully.

In addition, limited by low tumor immunogenicity and insufficient T-cell infiltration in the tumor area, only a small number of patients show positive treatment feedback towards a single ICB therapy [274, 275]. How to improve the immunogenicity of tumors and reverse immune “cold” tumors to immune “hot” tumors to compensate for the shortcomings of ICB therapy has become a research hotspot in the field of tumor immunotherapy in recent years [276, 277]. Recently, our group developed a cell membrane camouflaged CM@MnO_x_ nanozyme that mimics the activity of multiple enzymes in tumor, which combined with anti-PD-1 monoclonal antibody for tumor catalytic immunotherapy (Fig. 19b) [273]. The treatment strategy has the following characteristics: (1) In TME, ·OH and O_2_^·^^−^ produced from the inherent POD-like and OXD-like activities of CM@MnO_x_ can kill tumor cells and cause the death of immunogenic cells, meanwhile, the production of oxygen from the CAT-like activities can alleviate tumor hypoxia, thereby alleviating the tumor immune suppression microenvironment. (2) The released Mn^2+^ in TME further induces M1 repolarization of tumor-associated macrophages and DC cell maturation by activating the cGAS-STING pathway. (3) Under the synergistic effect of PD-1, CM@MnO_x_ can treat primary and metastatic breast cancer, transform the TME from “cold” to “hot”, and produce long-term immunological memory effect.

Given the above synergistic theranostic strategies, we can preliminary explore the key elements and necessary criteria of nanozymes for in vivo application. First, the elemental composition must be safe for organisms. Second, the size and morphology should be regulated since the catalytic reaction mainly occurs on the surface and is influenced by the exposed lattice structure, specific surface area, and morphology. For example, the catalytic activity commonly increased with the decreased size [47]. Moreover, the size and morphology of nanozymes must fit the requirements of in vivo safety, administration, targeting, metabolism, and so on. Third, surface modification strategies should be considered, because they not only affect biological effects such as the administration and metabolism of organisms, but also have a significant impact on the catalytic activity of nanozymes. Accordingly, surface coating and modification via macromolecules, PEG, and small biomolecules should be studied. Based on the above discussion, in our opinion, Fe_3_O_4_ and hydrotalcite nanozymes with suitable modifications have the potential to truly achieve clinical conversion due to their well-reported safety and good catalytic activity.

## Outlook

In recent years, nanozymes have attracted widespread attention from researchers due to their high catalytic activity, mild reaction conditions, good stability, and the physical and chemical properties of nanomaterials. Due to the unique electronic structure on surface, metal oxide nanozyme has not only rich enzyme activity but also magnetic, optical, and dielectric properties, which has been widely used in the diagnosis and treatment of diseases. Metal-based nanozymes induce oxidative stress to kill tumor cells by producing ROS through OXD-like or POD-like activities, and also catalyze H_2_O_2_ decomposition to produce O_2_ in the TME through CAT-like activities to alleviate hypoxia in tumor tissue. People can transport nanozymes to the corresponding microenvironment of cells based on the needs of experimental purposes to play a specific role. Therefore, based on the unique physiological environment and metabolites of TME, designing multifunctional nanozymes with tumor-specific treatment and diagnostic functions has great development prospects and application prospects. Future research might be carried out based on the following aspects.

1. The catalytic mechanism and key catalytic sites of nanozymes require in-depth analysis. At present, most research on nanozymes focuses on optimizing catalytic activity, with less attention paid to the analysis of catalytic mechanisms. Besides, except for a few single-atom nanozymes, very little about the key structural information of the catalytic sites of most nanozymes was revealed.
2. Further study on DFT and mechanical learning is needed for predicting and evaluating the activities of nanozymes. Although theoretical calculations and other methods were used to predict the catalytic pathway of nanozymes, few studies were able to explain the catalytic mechanism and structure–activity relationship of nanozymes combined with experimental verification.
3. Nanozymes usually possess multienzyme activities, while the relationship, mutual influence, enhancement or inhibition methods, and causes of different enzyme activities need to be addressed. How to improve the selectivity of nanozyme to catalyze a specific substrate or a class of analogues? The study will be of great significance for application fields that require one enzyme activity.
4. For cancer diagnosis and treatment, it is very important to reveal the relationship between the TME and the catalytic efficiency and selectivity of nanozymes. Furthermore, the activity and selectivity regulation of nanozymes in a biological environment should be revealed, because the activities of most nanozymes will be heavily shielded in the biological environment. Up to date, intrinsic structural engineering (*e.g.*, hybrid and defect engineering) and external triggers (*e.g.*, temperature, ultrasound, light, and small molecules) have been used for activity regulation of nanozymes in the biological environment. However, in-depth and detailed regulation strategies still need to be developed.
5. The biosafety of nanozymes needs further exploration. On the one hand, similar to other nanomaterials, toxicology studies including long-term cytotoxicity, administration, metabolism, excretion, biodistribution, pharmacokinetics, pharmacodynamics in larger animals, and in vivo experiments are still unconfirmed. On the other hand, for the catalytic therapy effect, the targeting, selectivity, specificity, in vivo activity, and localization of nanozymes remained unclear. The in vivo microenvironment is complicated, thus, the catalytic pathway of nanozyme in vivo is often uncontrollable. Meanwhile, the potential biocatalytic reaction in vivo and the mechanism at the biological interface are also difficult to predict, all of which will become factors affecting biological safety. Therefore, in the next stage, systematic research and analysis on the in vivo stability, pharmacokinetics (such as uptake, distribution, and metabolism), duration of therapeutic effects, and toxicity (such as hemolysis and coagulation analysis) of nanozymes should be revealed.
6. As summarized above, synergistic theranostic strategies of nanozymes were well reported, while the mutual influence, promotion, and synergistic mechanisms between different treatment modes need in-depth exploration and validation by using new technologies and means.
7. Repeatability and stability of nanozymes and the catalytic activity should be studied. Most reports are focused on the discovery of new nanozymes, while, there are very few reports on repeated validation on stability and other issues. The resolution of these issues is expected to accelerate the industrial application of nanozymes.
8. Multiple omics analysis methods to elucidate the molecular mechanisms need in-depth exploration. Multiple omics analysis at different molecular levels, such as genome, transcriptome, proteome, interaction group, epigenome, metabolome, liposome, and microbiome, to obtain relevant omics data and gain a deeper understanding of the regulation and causal relationships between various molecules in the treatment process should be devoted.
